# Understanding the Novel Approach of Nanoferroptosis for Cancer Therapy

**DOI:** 10.1007/s40820-024-01399-0

**Published:** 2024-05-02

**Authors:** Afsana Sheikh, Prashant Kesharwani, Waleed H. Almalki, Salem Salman Almujri, Linxin Dai, Zhe-Sheng Chen, Amirhossein Sahebkar, Fei Gao

**Affiliations:** 1 Department of Pharmaceutics, School of Pharmaceutical Education and Research, Jamia Hamdard, New Delhi, 110062 India; 2 Department of Pharmacology and Toxicology, Faculty of Pharmacy, Umm Al-Qura University, Makkah, Saudi Arabia; 3 Department of Pharmacology, College of Pharmacy, King Khalid University, 61421 Asir-Abha, Saudi Arabia; 4 State Key Laboratory of Southwestern Chinese Medicine Resources, Pharmacy School, Chengdu University of Traditional Chinese Medicine, Chengdu, 611130 People’s Republic of China; 5 Department of Pharmaceutical Sciences, College of Pharmacy and Health Sciences, St. John’s University, New York, 11439 USA; 6 Biotechnology Research Center, Pharmaceutical Technology Institute, Mashhad University of Medical Sciences, Mashhad, Iran; 7 Applied Biomedical Research Center, Mashhad University of Medical Sciences, Mashhad, Iran

**Keywords:** Ferroptosis, Nanoparticles, Ferrotherapy, Cancer, Genes, Nanotechnology, Toxicity, Reactive oxygen species

## Abstract

As a new form of regulated cell death, ferroptosis has unraveled the unsolicited theory of intrinsic apoptosis resistance by cancer cells. The molecular mechanism of ferroptosis depends on the induction of oxidative stress through excessive reactive oxygen species accumulation and glutathione depletion to damage the structural integrity of cells. Due to their high loading and structural tunability, nanocarriers can escort the delivery of ferro-therapeutics to the desired site through enhanced permeation or retention effect or by active targeting. This review shed light on the necessity of iron in cancer cell growth and the fascinating features of ferroptosis in regulating the cell cycle and metastasis. Additionally, we discussed the effect of ferroptosis-mediated therapy using nanoplatforms and their chemical basis in overcoming the barriers to cancer therapy.

## Highlights

- Nanoferroptosis: a novel cell death process using various nanoparticles.
- Therapeutic potential of nanoferroptosis inducers in cancer.
- Synergistic approach of nanoferroptosis with immunotherapy, sonodynamic and photodynamic therapy.

## Graphical abstract

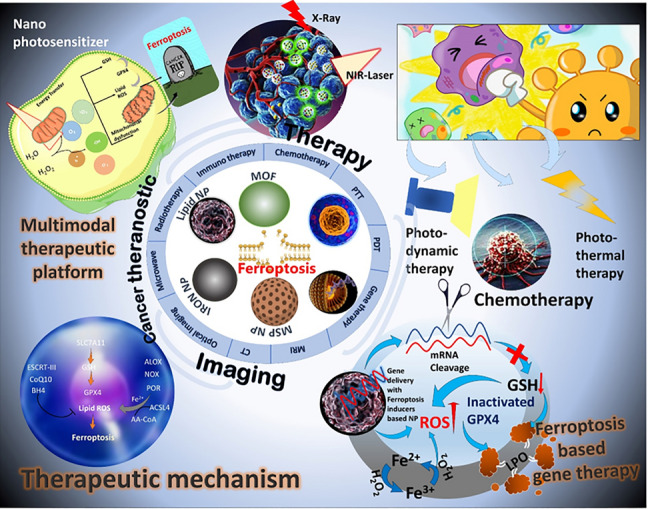

## Introduction

A new method of cancer cell death apart from apoptosis, autophagy and necrosis has been discovered a decade ago. Such a method was termed “ferroptosis” which is an iron-mediated/dependent non-apoptotic but regulated cell death (RCD), specifically for those cells overexpressing small mutated GTPase [1–3]. Apoptosis is the primary mechanism behind drug therapy which has made a significant contribution to cancer therapy [4, 5]. However, the heterogeneous and mutating nature of cancer cells develops mechanistic resistance to prevent apoptosis. Since cancer continues to be a major health issue, which had cost millions of lives, thus it became exceedingly vital to investigate additional types of cell death mechanisms to overcome medication resistance [6–8]. This recently identified type of cell death called ferroptosis, which is exploited in cancer therapy, is best described by aberrant intracellular reactive oxygen species (ROS) build-up [3, 9]. In cancer cells, ferrous iron (Fe^2+^) has a high metabolic and catalytic activity. After deactivating glutathione peroxidase 4 (GPX4) in cells, ferroptosis raises ROS accumulation and inhibits cellular anti-oxidant defense mechanism, thereby causing cellular structure damage and mitochondrial inhibition [10, 11]. In addition of ferroptosis, other major types of cell death [12–16] are summarized in Table 1. The major strength of ferroptosis is the technique for cell death that avoids multi-drug resistance (MDR) and improves traditional chemotherapy, demonstrating the excellent therapeutic effect. Ferroptosis is crucial for survival as well as the death of malignant lesions. Cancer cells are thought to be particularly vulnerable to ferroptosis in the general mode because of their active oxidative metabolism and rapid cell cycle. The regulation of ferroptosis in cancer cells has also been linked to other conventional cancer-related genes, including p53 [17]. The combination of numerous trials targeting the ferroptosis inhibitory protein can effectively prevent the growth of cancer cells in vitro, although some cancer cell lines increase the ferroptosis inhibitory protein to develop drug resistance. Ferroptosis is also tightly associated with the availability of accessible ferrous ions (Fe^2+^). Divalent metal transporter 1 (DMT-1) present in the endosome reduces ferric iron (Fe^3+^) to Fe^2+^, which participates in the electrochemical Fenton reaction with hydrogen peroxide (H_2_O_2_) and produces deadly ROS. As a result, the Fenton reaction is crucial in initiating tumor-specific ferroptosis. Thus, in the biomedical field, judicious application of such characteristic mechanisms can control the cell cycle and tumor-specific ferroptosis induction has been viewed as a possible approach for anticancer therapy [18].

**Table 1:** Representation of characteristic features of various forms of cell death

Type of cell death	Characteristic features
Apoptosis	It is a programmed cell death (PCD) wherein both the nuclear and cellular size decrease, leading to the fragmentation of nuclear bodies while maintaining an unchanged response to the mitochondria
Ferroptosis	A newly discovered regulated form of cell death that damages mitochondrial Crista and ruptures the nucleus and mitochondrial membrane
Necroptosis	This kind of PCD causes rupture of cell membrane, random DNA degradation, swelling of cell and its deformation and deformation of organelles
Cuproptosis	Aggregation of lipoylated mitochondrial proteins

Other than cell death, ferroptosis also deals with cancer immunotherapy [19, 20]. Robust immunogenicity facilitated by ferroptosis-mediated apoptotic cell fragments can effectively bring the immune system and elicit antigen-specific immunity to the neoplasm. However, due to the escape mechanism of the tumor immune system, immunotherapy for the therapy of solid tumors showed relatively low therapeutic efficacy. Ferroptosis witnessed extraordinary benefits in cancer response; however, the existing approaches to encourage ferroptosis were mostly simplistic and ineffective. In reality, the multiplying cancer cells generate glutathione (GSH) abundantly which consumes the endogenous ROS. Moreover, due to the limited capacity of cancer cells to generate sufficient iron ions, depleting GSH becomes a more challenging process [21]. Thus today, multifunctional, targeted and effective nanocarriers for carrying drugs and ferroptosis inducers or imaging agents to the site of the application are highly demanded [22].

Nanomedicine has garnered specific attention owing to its exclusive properties [23–30]. Nanostructures are being focused on the delivery of pharmaceuticals, nutraceuticals and genes along with numerous antibodies [23, 31–38]. The unique architecture of such structures makes their supply more feasible and efficient with minimum to no side effects. Nanomaterials offer a compelling substitute for small molecule drugs, given the advantages arising from their distinctive structures. Initially, their small size enables nanomaterials to precisely target cancer cells or tissues through passive means, leveraging the enhanced permeability and retention (EPR) effect. Alternatively, they can achieve active targeting by attaching to specific ligands like aptamers and antibodies. The nanoparticle development featuring distinct functional characteristics, including photothermal effects, photodynamic effects, imaging capabilities and magnetic hyperthermia effects, presents an opportunity to construct multifunctional theranostic nanoplatforms for cancer management. These properties complement traditional theranostic agents, enhancing the potential for comprehensive cancer diagnosis and treatment. However, the major drawback associated with nanomaterials is immunogenicity, biodegradation and biocompatibility issues which are still needed to be addressed. What is being observed with chemotherapeutics is their short half-life and high rate of toxicity, which have a minimal therapeutic effect on desired site while causing multiple toxic reactions in the other part of the undesired site. Such a deleterious response reduces the therapeutic value and hence survival of cancer patients. The aim of therapy for malignancy or even other diseases should be based on a high therapeutic response rate with minimal side effects. Nanoparticles show some hope for clinicians and researchers in overcoming such aforementioned hurdles. For example, Jiang et al. in a recently established ferroptosis-mediated cancer cell death using erastin, rapamycin and Fe^2+^ based micelle [39], while in another study by Tang and team, sorafenib-loaded manganese oxide nanoparticle depleted the GSH consumption and inactivated GPX4 causing intracellular elevation of lipid peroxide and finally death of hepatocellular carcinoma cells by ferroptosis [22]. The field of nanotechnology has tremendous scope in the development of biomedicine as scientists and researchers are continuously working in such areas, bringing multiple fascinating nanoparticles into reality. One such nanoparticle developed was the Janus nanoparticle (JNP). The anisotropic structure of nanosystems has received prodigious value owing to their multichambered tunable configuration which exhibited several functions such as bioimaging, cell targeting and drug delivery. Zhu et al. employed sorafenib-based “ball-rod” JNP and protected it with tannic acid to facilitate blood circulation against non-small cell lung cancer (NSCLC). Sorafenib, on one hand, downregulated GPX4, while TA boosted the Fenton reaction. These multiple functions in one pot reinforced ferroptosis to induce cell death [40]. Ferroptosis is not one-step mechanism but involves different associated factors which have been discussed in the sections below. However, one should also understand the importance of iron to induce cell growth and its progression to finally understand the actual need of ferroptosis. The inducers of ferroptosis (Table 2) have to first reach the targeted site to induce ferroptosis. Nanoparticles enable their cellular uptake, avoid lysosomal uptake, promote pH-based release, extend the release rate, improve blood circulation and promote their therapeutic window. Thus, we suggested the term “nanoferroptosis,” which means nanoparticles mediated ferroptosis. Reviews published previously entail the biomedical scope of ferroptosis, entailing the process and treatment strategies in various disease [41]. We would like to appreciate various other reviews wherein nanoparticle mediated ferroptosis such as Refs. [42–45]. Literature on vulnerability of cancerous cells toward ferroptosis, stimuli-responsive ferrotherapy, biology and mechanism of ferroptosis and photonic nanomaterial-based cancer therapy has already been discussed [46–49].

**Table 2:** Kinds of ferroptosis inducers

Inducers of ferroptosis	Function	References
Doxorubicin	HO-1	[251]
RSL3	GPX4 inhibition	[252]
Hemin	Iron accumulation	[253]
Sulfasalazine	Inhibition of System Xc^−^	[254]
Altretamine	GPX4 inhibition	[255]
Glutamate	Inhibition of System Xc^−^ that affect cellular transport of cysteine	[256]
FINs	ROS generation	[10]
Bromelain	ROS generation in KRAS mutation	[257]
All-trans retinoic acid (ATRA)	ROS generation	[258]
Lapatinib	Improve oxidative stress inside the cells	[259]
Artesunate	Induces programmed cell death through ROS generation	[260]
Ferumoxytol	Lipid peroxidation	[261]
Cotylenin A	ROS generator	[262]

However, a detailed explanation regarding the molecular pathway (with special emphasis on current/ongoing research) has not been established successfully. Furthermore, from chemistry perspectives, this review presented adequate knowledge on designing of nanoparticles for inducing apoptosis. Our aim was to fill the gap and bring forth up-to-date information under the microscope of scientists, oncologists and researchers for better understanding of topic. The review has summarized the recent finding of actual prerequisite of ferroptosis using nanoparticles in abstaining cancer cells and MDR along with the employment of genes for cancer therapy.

## Elucidating the Importance of Iron in Cancer Progression

Iron plays a significant role in a variety of biological processes as a primary inorganic nutrient, including synthesis and replication of DNA and RNA, aerobic respiration (e.g., cytochrome, cytochrome c oxidase, Rieske protein and ferredoxin), oxygen transport, functioning among several enzymes, synthesis of heme, immunological functions, detoxification procedures, metabolism and iron-dependent signaling [50, 51]. Additionally, iron is required for the generation of iron-sulfur clusters (ISC) and heme, which upon integration into proteins execute numerous vital processes such as oxidative phosphorylation and citric acid cycle [52, 53]. In healthy cells, iron homeostasis is strictly controlled by a fine balance between cellular uptake and storage, systemic transit and absorption. Conversely, perturbation of this equilibrium has been attributed to carcinogenesis and may raise the risk of cancer. Extensive research addressed iron control pathways and the connection between elevated tumor growth with increased iron content. In particular, macrophage deposits in cancerous and metastatic cells of the breast, lung and brain were shown to include high-iron clusters that harbored hemosiderin. At the cellular level, a complex formed after the binding of iron with transferrin (TF) is identified as transferrin receptor 1 (TFR-1) present on the cell membrane. This complex undergoes endosomal endocytosis wherein Fe^3+^ in presence of iron reductase reduces to Fe^2+^ primarily by members of the family of six-transmembrane prostate epithelial antigens (STEP1-4). Following that, the cytoplasmic labile iron pool (LIP), which is principally mediated by divalent metal-ion transporter 1 (DMT1), transports iron in the cytosol [54–56]. For several metabolic purposes, this newfound metabolically active iron can be transported to various cell compartments or can be stored in ferritin (a complex of protein that accumulates iron in its inactive state). Ferroportin, the only known cellular iron efflux pump, allows the elimination of the excess iron from the cell which works in conjunction with hephaestin or ceruloplasmin for maintaining cellular iron homeostasis. In this regard, the system of iron-responsive elements-iron regulatory proteins (IREs-IRPs) modulates iron levels resulting in ferroportin degradation to achieve homeostasis, while the circulating hormone, hephaestin, upholds homeostasis at the systemic level. Several studies have shown the association between aberrant iron metabolism and other human disorders, especially carcinoma. Tumor cells can simultaneously upregulate antioxidant defenses for survival, for instance, antioxidant transcription factors activation and escalating the articulation of anti-oxidant genes, because their rate of proliferation is typically higher than those observed in healthy ones, so is their demand for iron. This results in exceeding oxidative stress. On the other hand, since iron is crucial for the proliferation and growth of tumor cells, they are more sensitive to its reduction than healthy cells. Increased iron metabolism, its input and affinity, combined with suppression of its output, are the primary ways that this imbalance in cancer manifests itself, leading to iron build-up. Research indicates that characteristics of cancerous lesions in cholangiocarcinomas and breast appear to be closely related to increased heavy-chain ferritin (H-ferritin) expression. Except for TFR1, levels of proteins involved in iron trafficking in various malignancies are a topic of debate related to cancerous and non-cancerous cells [57, 58]. An overexpression of TFR1 has been frequently observed in the tissues and cancer cells from leukemia, glioma, ovarian, prostate, breast, liver and colorectal malignancies. Iron plays a critical role in stem cell behavior by serving as a co-factor for epigenetic enzymes like TET enzymes and proteins with the JmjC domain. By using iron-mediated epigenetic mechanisms, cancerous cells modify the Wnt, canonical Notch and hedgehog signaling pathways for their self-renewal and maintenance [59, 60]. When considered collectively, some of the key proteins in metabolism of iron, including their protein ferritin iron transporter TF and its receptor, the iron regulator hepcidin, the iron exporter FPN1 and epigenetic enzymes, may offer therapeutic hope for the treatment of cancer.

## Features of Ferroptosis

The term ‘ferroptosis’ was proposed a decade ago by a scientist in USA named Dixon [3]. Unlike apoptosis, necrosis and autophagy, ferroptosis is an iron and ROS-dependent cell death having altered cytological characteristics, including deformed and ruptured outer mitochondrial membrane and reduced mitochondria cristae [61]. Such activities are due to the loss of plasma membrane permeability under oxidative stress and membrane lipid peroxidation. However, the nucleus remains in normal size; the cell membrane remains intact with no observable chromatin concentration [48, 62]. Biochemically, due to reduced activity of GPX4 and depletion of GSH, the lipid peroxides could not be metabolized causing oxidation of lipids by Fe^2+^ in a Fenton alike manner, leading to accumulation of ROS that promotes ferroptosis [48]. Genetically, it was found that erastin, which is a prototype of ferroptosis inducer, blocks the uptake of cysteine (cys) by directly inhibiting system Xc^−^, which in turn promotes GPX4 degradation by encouraging chaperone-mediated autophagy. Another category that inhibits GPX4 activity includes DP17 and RSL3. System Xc^−^ is the glutamate/cysteine antiporter that imports cystine into the cell. Herein, cystine converts to cysteine which promotes GSH synthesis. GSH acts as a substrate for GPX4 that converts hydrogen peroxide into water, safeguarding the cell from oxidative stress. GPX4 also converts toxic lipid peroxides into non-toxic lipidic alcohols [63]. In a recent study, chemotherapeutic agents RSL3 and ferrocene were combined to improve anti-cancer and anti-metastatic effect through synergistic response of therapy by apoptosis as well as ferroptosis [64].

Fundamental evidences suggested that the induction of ferroptosis does not merely rely over oxidative stress as those promoted by GSH synthesis or cystine/glutamate antiporter system but also the metabolism of unsaturated fatty acids and iron [65]. Under such conditions, ferroptosis has been evidenced in various pathological conditions such as varied renal diseases (polycystic kidney disease and acute kidney injury), neurodegenerative disorders (Parkinson’s and Alzheimer’s diseases), ischemic stroke, brain damage and cancers [66, 67]. Specifically, the higher sensitivity of malignant cells toward ferroptosis has been an additional advantage in cancer treatment especially those with inherent drug resistance. Ferroptosis is also associated with cancer immunotherapy [68]. Recent studies establish the considerable role of ferroptosis in cancer cell regulation which could fill a huge gap in cancer therapy.

## Understanding the Molecular Basis of the Ferroptosis Process

The underlying feature responsible for ferroptosis is lipid peroxidation which involves the imbalance between the intracellular antioxidants and the intracellular free radicals. Such imbalanced conditions cause oxidative damage to the cellular membrane leading to lipid peroxidation, accumulation of iron ions and finally cell death [69].

### Suppression/Inhibition of GPX4

**Fig. 1:**
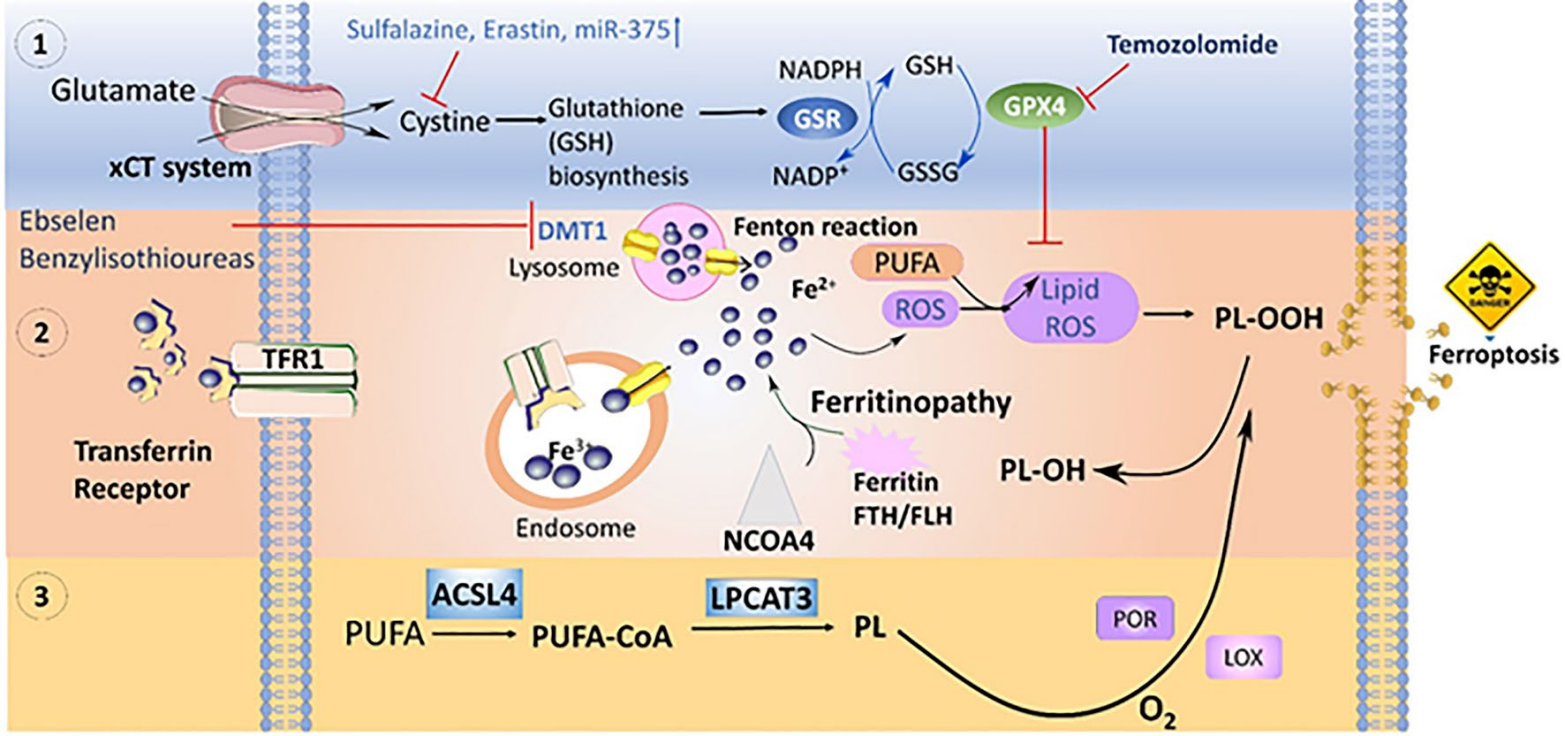
Representation of the ferroptosis mechanism in cancer therapy. Three pathways are primarily associated; inhibition of GPX4, iron metabolism and lipid peroxidation. **1** Erastin, which is a prototype of ferroptosis inducer, blocks the uptake of cysteine (cys) by directly inhibiting system Xc-, which in turn promotes GPX4 degradation by encouraging chaperone-mediated autophagy, **2** increase in LIP downregulate DMT1 to promote oxidative stress causing ferroptosis, **3** recombinant lysophosphatidylcholine acyltransferase 3 (LPCAT3) and Acyl-CoA synthetase long-chain family member 4 (ACSL4) are the catalysts required for the synthesis of PUFA-containing phospholipids which under oxidative stress promotes ferroptosis

GPX4 is a selenoprotein, initially discovered by Ursini and team through a biochemical purification technique [70]. To discover small anti-cancer molecules, Stockwell and team, in the year 2001, used a high-throughput screening technique that led to a publication in 2003, illustrating the number of compounds possessing non-necrotic and non-apoptotic cell death characteristics. After the counter screening, it was found that hydrophobic radical scavenging antioxidants and iron chelators repressed such cell death mechanisms. With further mechanistic investigations, two cellular components were identified, namely, GPX4 and system Xc^−^, which on suppression causes ferroptotic death [10]. GPX4, thus, is a prominent target for grueling neoplastic cells specifically those involved in therapy resistance. RSL3 inactivates GPX4 after binding with it leading to the accumulation of ROS. Chemically, RSL3 contains a chloroacetamide and electrophilic moiety, which reacts with the nucleophilic amino acid residues of GPX4. Mainly, selenocysteine (nucleophilic amino acid residue) drives a link between the active sites of GPX4 and RSL3 [71]. Ferroptosis of colorectal cancer cells (CRC) was investigated for the first time by Sui et al. The cells after treatment with ferroptosis inducer RSL3 showed dose and time-dependent cell death due to increased cellular labile iron pool and ROS level accompanied by reduced GPX4 expression (Fig. 1) [72].

Considering CRCs, nearly 36%–46% of cases show KRAS mutation, which fails to demonstrate a considerable therapeutic effect of cetuximab. As already observed from the previous study, RSL3 demonstrates ferroptosis by downregulating the GPX4 level. This shed the light on the link between KRAS mutant CRC and ferroptosis. It is worth mentioning that cetuximab is a regulator of p38 mitogen-activated protein kinase (p38 MAPK), which could suppress Nrf_2_/HO-1. Considering such factors, Yang and the team assessed the effect of cetuximab in combination with RSL3 on viability of KRAS mutant CRC cells. The combination therapy reduced Nrf_2_/HO-1 expression as evidenced by western blotting results. An elucidated expression of malondialdehyde with accrual lipid reactive oxygen species was also observed which is due to cetuximab that promoted RSL3-mediated ferroptosis. Accordingly, such treatment strategies could further be investigated with chemotherapeutics and gene silencing agents to have potential therapeutic approaches [73].

In another study, the effect of propofol was determined on cisplatin-resistant non-small cell lung cancer (NSCLC). Propofol elevates the expression of miRNAs having tumor-suppressive effect. Based on the results, propofol upregulated miR-744-5p/miR-615-3p by inhibiting GPX4 transcription. Consequently, propofol reduced cisplatin resistance and tumor growth in vivo by inducing ferroptosis [74]. Another compound, trabectedin, irrespective of p53 status, exhibited cytotoxic effects on NSCLC by upregulating ROS, iron and lipid peroxidation causing ferroptosis [75].

Bufotalin an active constituent of bufadienolide is a traditional Chinese medicine that was extracted from *Venenum bufonis.* It is a toxic steroid with demonstrable anti-cancer activities. In this context, a study by Zhang et al. showed the anti-proliferation of A549 cells due to the induction of ferroptosis by bufotalin. The compound increased intracellular Fe^2+^, inhibited the protein expression of GPX4 and elevated its degradation. Furthermore, ubiquitination of GPX4 was also induced by bufotalin as demonstrated by immunoprecipitation assay. Thus, the compound could serve as an important candidate in cancer therapy by selectively elevating the level of Fe^2+^, causing lipid peroxidation and GPX4 degradation and finally inducing ferroptosis [76]. Regulation of GPX4 level, ROS generation and lipid peroxidation are evident in numerous studies. Recently, a flavonoid, Icariside II (IC II) snatched the eye of the research group in China that responded toward renal cancer cells through the induction of ferroptosis by downregulation of GPX4. Additionally, IC II upregulated miR-324-3p that suggests GPX4 knockdown [77]. Apart from cancer, ferroptosis also demonstrates a critical role in the manifestation and progress of several diseases such as neurodegenerative diseases and acute organ failure. Ferritin being a cytoplasmic protein exhibited versatile functions including immunoregulation and storage of iron [78–80]. Another novel ferroptosis inducing compound which acts through the degradation of GPX4, discovered was ferroptosis inducer-56, also called as FIN56 [81]. This compound emerged from the screen of caspase-independent lethal (CIL) compounds, induces GPX4 degradation and also acts through mevalonate pathway by exhausting coenzyme Q10 (CoQ10). Chaperon-mediated autophagy also increases the sensitivity to ferroptosis contributed by degradation of GPX4 [82].

### Lipid Peroxidation

Ferroptosis is extensively connected with lipid metabolism owing to its peroxidation and biosynthesis. The peroxidation of membrane lipid produces phospholipid hydroperoxides, which after decomposition forms malondialdehyde or 4-hydroxynonenal. Such decomposed residues enhance membrane permeabilization and instability leading to cell death [83]. Noteworthily, the proclivity of lipid enduring peroxidation relies upon the asset of carbon-hydrogen bond, as during the peroxidation process, specific carbon loses hydrogen to attach with peroxyl group (O–O). Polyunsaturated fatty acids (PUFA) have been proclaimed since their discovery about being peroxidation susceptible owing to the presence of weak carbon-hydrogen single bonds (C–C) in between two carbon–carbon double bonds (C=C) structures, suggesting PUFAs as key derivatives of ferroptosis (Fig. 1) [84]. A study by Zou et al., suggested the role of cytochrome P450 oxidoreductase (POR) in the peroxidation of PUFAs causing ferroptotic cancer cell death. Using systematic lipidomic profiling and assessing genetic depletion of POR, it was revealed that POR accelerates ferroptosis on comprehensive range of lineages and cell states [85]. Lipid peroxidation generated ROS especially hydroxy ions (·OH) which interact with PUFAs; ·OH is also the main source of Fenton reaction (reaction in between Fe^2+^ and hydrogen peroxide). Arachidonic acid (AA) and linoleic acid (LA) are used as substrate of lipoxygenases (LOXs) which catalyses di-oxygenation of esterified as well as free PUFAs delivering several lipid hydroperoxides [86, 87].

Cancer cells demand lipid uptake providing energy for their growth and survival [88, 89]. Furthermore, lipid metabolism in cancer cells delivers sufficient protection against lipid peroxidation by developing lipid desaturation and lipid droplets. Lipid droplets are the organelles overexpressed in cancer cells and are storage house of lipids, which under elevated conditions are associated with stemness of cancer lesions [90]. Thus, cells could counterattack ferroptosis by avoiding lipid peroxidation. Moreover, recombinant lysophosphatidylcholine acyltransferase 3 (LPCAT3) and Acyl-CoA synthetase long-chain family member 4 (ACSL4) are the catalysts required for the synthesis of PUFA-containing phospholipids. Depletion of such enzymes encoded genes averts entry of PUFA into phospholipidic membrane, thereby inhibiting the development of PUFA-containing phospholipids, its peroxidation making cells less sensitive to ferroptosis [91].

Numerous studies conducted in past culminated evidences of ferroptosis-mediated cell death through lipid peroxidation. Apatinib, the first angio-genic agent approved by cFDA, is a selective inhibitor of vascular endothelial growth factor receptor-2 (VEGFR2) tyrosine kinase approved for the treatment of gastric cancer. It has shown progressive improvement as a second-line therapy for the advanced gastric cancer patient taking platinum or fluoropyrimidine-based therapies. Since ferroptosis is different from necrosis or apoptosis, it remains unknown whether lipid peroxidation under the process of ferroptosis is mediated by apatinib. To undermine, Zhao and co-workers investigated the apatinib effect in various gastric cancer and normal cell lines, with further exploring the inhibitory potential of antioxidant defense enzyme GPX4 on lipid ROS production, GSH level, cell death, cell viability, protein expression and cellular malondialdehyde (MDA) levels. Apatinib reduced the GSH level while elevating lipid peroxidation levels to induce ferroptosis. However, such effect was hindered by liproxstatin-1, vitamin E, ferrostatin-1 and GSH. Apatinib also repressed transcription factors Sterol regulatory element-binding protein-1a (SREBP-1a) causing downregulation of GPX4. Additionally, multi-drug-resistant gastric cell also responded toward apatinib therapy due to inhibition of GPX4 [92]. Exploiting the lipid peroxidation process, Jiaqi and team showed the metastasis and proliferation restraining property of andrographolide (AD) in NSCLC cell lines. Ferroptosis initiation was validated by ROS reduced GSH expression, elevated level of malondialdehyde (MDA), GSH and ROS. Synchronously, AD intensified dysfunction of mitochondria, as revealed by mitochondrial membrane potential (MMP) depolarization, elevated increased mitochondrial ROS release and reduced mitochondrial ATP [93]. Intervention in redox balance and lipid metabolism has shown to encourage ferroptosis in numerous cancers. However, ferroptosis is a gradual phenomenon regulated by multiple metabolic pathways, a clear picture of which is still unrevealed in cancer therapy. Flipping the coin unveils three defense mechanisms of cells against ferroptosis such as coenzyme Q10 (CoQ10) system, GSH system and thioredoxin (TXN) system which suppress ferroptosis by detoxifying lipid hydroperoxides. A recent study demonstrated ferroptosis landscape of triple-negative breast cancer (TNBC) illustrating the combinatorial effect of anti-PD1 and GPX4 in inducing tumor ferroptosis with augmented anti-tumor immunity [94].

Likewise, such observations clarify that the process of ferroptosis depends of ROS accumulation, lipid peroxidation and GPX4 retardation to reinforce therapeutic system. The medical system first needs to strengthen and study the underlying principle in cancer progression which once altered could serve the health of millions.

### Iron Metabolism

Iron serves as a crucial element in tumor progression and its recurrence, while a range of iron metabolites and related proteins are unusually regulated indicating an increased level of intracellular iron, which could be a metabolic hallmark in cancer cells. Heme, iron or iron clusters could bind with ROS-producing enzymes such as NOXs (NADPH oxidase), arachidonate lipoxygenases (ALOXs) and XDH (xanthine dehydrogenase) developing lipid hydroperoxides (a substrates for Fenton reaction). Additionally, cytochrome P450 oxidoreductase (POR) and 12-lipoxygenase are also required for ferroptosis. The sensitivity to ferroptosis is also controlled by kinase ataxia telangiectasia (ATM) by regulating ferroptosis abundance. Iron reacts with endoperoxide to trigger ferroptosis **(**Fig. 1**)**. Ferroptosis-inducing oxide (FINO_2_) is other class of ferroptosis inducers which does not inhibit system Xc^−^ and rather act on GSH inducing GPX4 degradation. Cells after treatment with FINO_2_ share portion of oxidized lipids, suggesting FINO_2_ endures a Fenton reaction forming alkoxyl radical to induce lipid peroxidation. FINO_2_ could also bind with iron-dependent enzymes or even with activated lipoxygenases to induce ferroptosis. In fact, FINO_2_ reacts with Fe(II) undergoing Fenton reaction to generate alkoxyl radical and finally results in lipid peroxidation. On contrary, FINO_2_ can also oxidize non-heme iron cofactor following binding or activation of iron-dependent enzymes or lipoxygenases [95]. Iron accumulation derives ferroptosis execution by ensuring dysfunctioning of system Xc^–^ (cystine/glutamate antitransporter) by deactivating GPX4 and generating Fenton chemistry between hydrogen peroxide and iron. A report mentioned development of cancer cell membrane-flagged iron-small interfering RNA nanohybrid for the therapy of cancer. The siRNA is against solute carrier family 7 member 11 (SLC7A11), an important cystine transporter of system Xc^–^ that affect cystine upregulation, thereby inhibiting the biosynthesis of GSH. As a result, iron encouraged ROS generation and siRNA depleted GSH, causing synergistic ferroptosis performance in cancer cells. Also, accumulation of iron permitted magnetic resonance imaging (MRI) which is a desirable feature for non-invasive monitoring of effective therapy. Overall, the one-pot strategy promised a great clinical response in cancer treatment [96]. Researchers are focusing more on combinatorial approach for synergistic performance of the therapy. Zhang et al. developed magnetosomes having a core made of Fe_3_O_4_ magnetic nanocluster coated with TGF-β inhibitor (Ti)-loaded leukocyte membranes. Leukocyte camouflage not only improves the circulation time but also enables Ti (hydrophobic) loading. PD-1 antibody was also decorated on the surface to make the magnetosomes bind with PD-1 receptor overexpressed cancer cell. Release of Fe from the core generated ROS via Fenton reaction causing lipid degradation. This is turn depleted GSH and GPX4 level causing cell ferroptosis. It is worth mentioning that ferroptosis is malignant cells also immunomodulate tumor microenvironment. An amalgamation of tumor cell-responsive agents in magnetosomes inflicted ferroptosis and immunomodulation for the high-performance cancer therapy [97].

## Chemical Basis of Nanoparticle-Induced Ferroptosis in Cancer Management

An equivalent but distinct strategy for treating tumors is ferroptosis therapy, which employs methods to produce ROS including ·OH, leading to the accumulation of lethal lipid peroxides, based on the significant polyunsaturated phospholipids peroxidation on the cellular membrane. Additionally, due to the redox equilibrium, GSH overexpression in the TME can scavenge the harmful ROS to protect against the cell damage produced on by oxidative stress and hence significantly reduce performance of ferrotherapy [98–100]. Actually, the formation of ROS begins with the iron-dependent Fenton reaction. Various nanoparticles have reported ferroptosis-based cancer therapy including metal organic framework, lipid-based nanocarrier, iron nanoparticles, etc. Iron oxide nanoparticles (IONP) convert H_2_O_2_ into ·OH and thus enable the cellular internalization through lysosomal pathway.

**Fig. 2:**
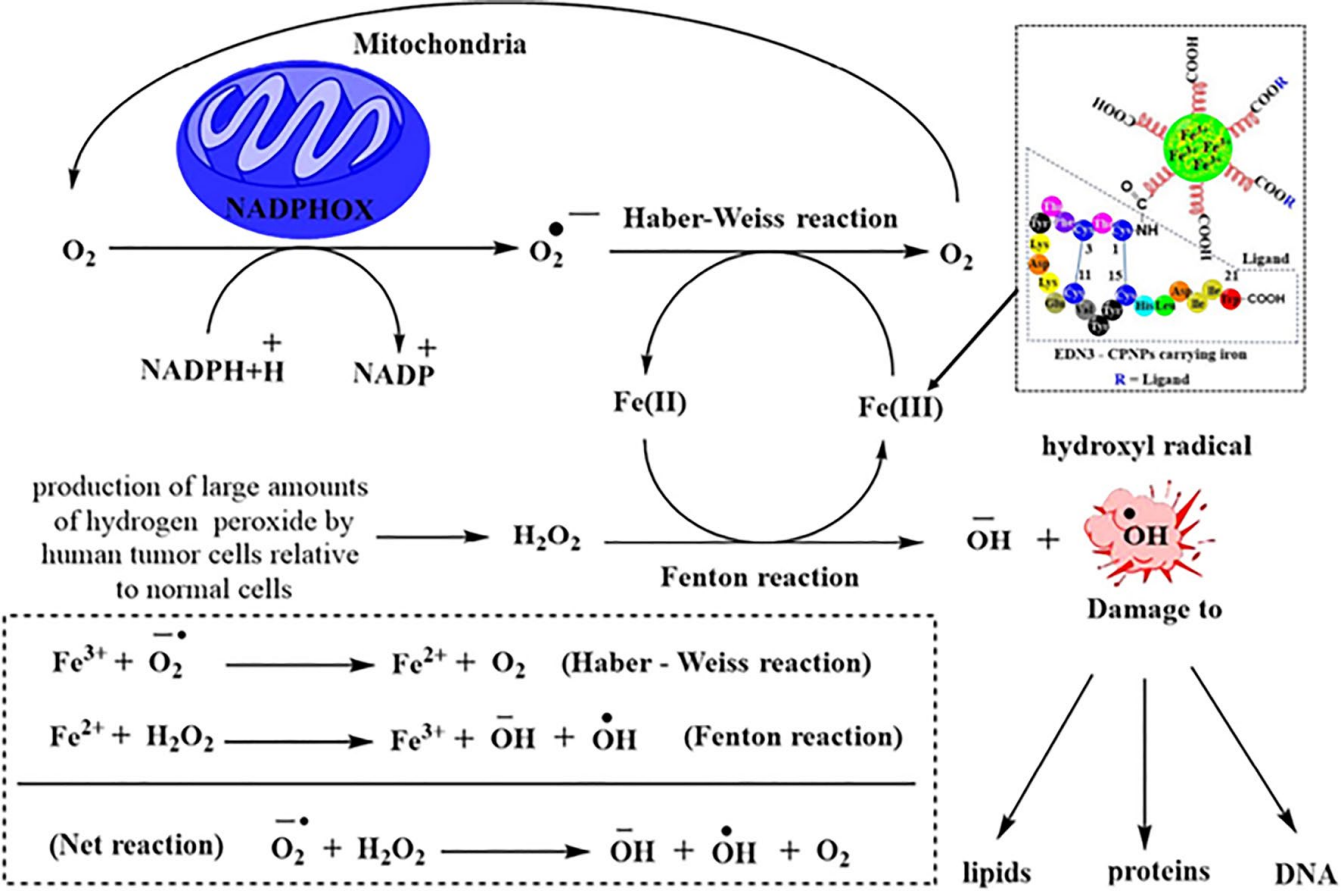
Representation of ferroptosis effect of endothelin-3 targeted nanoparticle in production of highly reactive hydroxyl radical [101]

Today, the aim of cancer therapy is not only to reduce proliferation but also to reduce toxicity. Iron-loaded endothelin-3 (EDN3) modified conjugated polymer nanoparticles (EDN3-CPNP) had catalyzed Fenton reaction besides targeting endothelin-B overexpressed melanoma cells. As shown in Fig. 2, the Fe^3+^ delivered by EDN3-CPNP was reduced to Fe^2+^ by Haber–Weiss reaction to undergo Fenton reaction, finally producing reactive hydroxyl radical and returning molecular oxygen as a product [101].

To exhibit a remarkable efficacy for therapy against tumors, copper-iron peroxide nanoparticles developed by Koo et al., self-supplied H_2_O_2_, released iron and copper ion maintaining the major portion at lower oxidation state. This in turn facilitated production of hydroxyl radical through an efficient catalytic loop. As a consequence, with a low treatment dosage, practically complete tumor ablation was exhibited without the need of any further therapeutic techniques [102]. As per our understanding, an important point required to be kept in consideration is tumor microenvironment’s hypoxic condition that derives the Fenton reaction. If the cells are less hypoxic, then agents like Fe have to be supplied along with nanocarriers to mediate ferroptosis-based cancer therapy.

**Fig. 3:**
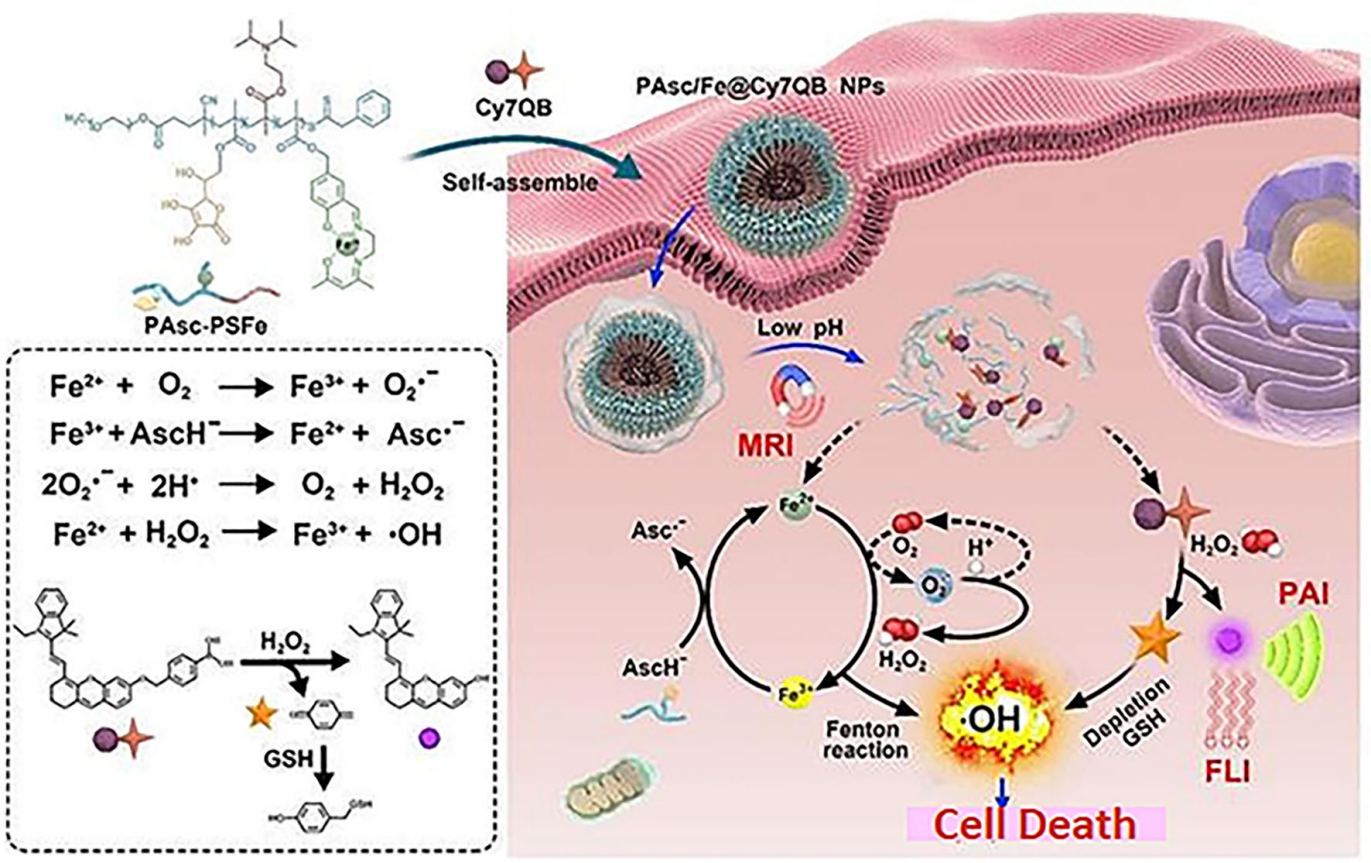
The cascade of cell death inducing mechanism of nanomicelles. The theranostic nanomicelles produced highly reactive hydroxyl radical by releasing of AscH^−^, Fe^2+^ and the adjuvant Cy7QB [103]

Self-assembled nanomicelles were developed in another study showed Fenton like reaction and imaging of solid tumors. Such a strategy would be cost-effective as well as tumor responsive. The theranostic nanomicelles were created by copolymerizing 2-(diisopropylamino) ethyl methacrylate (polymerized diisopropylamino, PDPA), pH-sensitive segments of Schiff base embedded with Fe^2+^ and ascorbate (polymerized ascorbic acid, PAsc). Due to its inherent hydrophobicity, this complex could be packed into a hydrophobic core, and it could turn hydrophilic once the Schiff base was broken down and the amine groups were protonated in an acidic environment. The newly formed H_2_O_2_-responsive probe (Cy7QB, a Cy7 dye combined with the quinone methide-producing boronate ester) was encapsulated inside the hydrophobic core of PAsc-PSFe to create the PAsc/Fe@Cy7QB nanomicelles. Following systematic delivery, the PAsc/Fe@Cy7QB nanomicelles attracted tumor cells' internalization and endocytosis via the increased permeability and retention (EPR) effect. Following the Schiff base's breakdown and protonation of PDPA, the acidic environment of endosomes and lysosomes released ascorbate monoanion (AscH^-^) free Fe^2+^, and the adjuvant Cy7QB into the cytoplasm. At this point, the release of Fe^2+^ coincided with a substantial enhancement in the Fe-mediated T1 magnetic resonance imaging (MRI) signal. Notably, the Fe^2+^ catalyzes the oxidation of AscH^-^ into the ascorbate radical (Asc^·-^) to produce H_2_O_2_, which could then be effectively transformed into highly active ·OH via the Fenton reaction along with the endogenous H_2_O_2_. Additionally, H_2_O_2_ might activate the adjuvant Cy7QB to produce the quinone methide (GSH-scavenger) and boosted the production of ·OH. In order to observe the process, the dye Cy7 that is simultaneously produced might be employed as an imaging agent for photoacoustic imaging (PAI) and fluorescence imaging (FLI). Due to their capacity to considerably increase the bioavailability of therapeutic drugs, effectively and precisely limit tumor development in vivo and have minimal side effects, stimuli-activatable chemodynamic therapies are therefore viable solutions for the treatment of tumors (Fig. 3) [103].

**Fig. 4:**
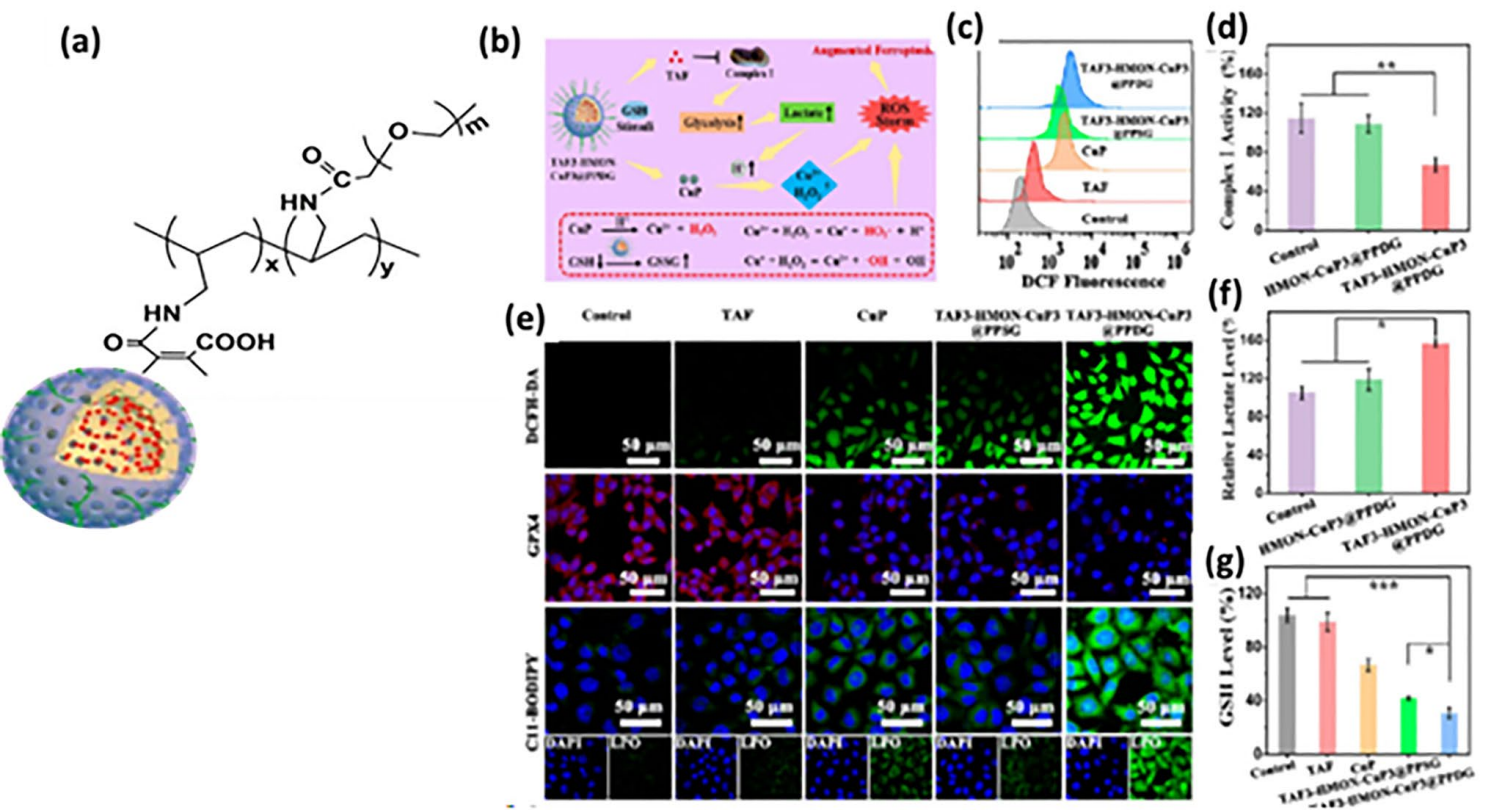
**a** pH-responsive polyethylene glycolpoly(allylamine)-dimethylmaleic anhydride (PEG-PAH-DMMA)-coated tamoxifen-loaded copper peroxide nanoparticle-capped hollow mesoporous organosilica nanoparticle (HMON), **b** illustration of ROS storm generation by developed nanoparticle,**c** lscm images showing GPX4, ROS and lipoxygenase level, **d** estimation of ROS level by flow cytometry in 4T1 cells laden mice, **e** relative complex I activity measurement and **f** lactate level measurement in 4T1 cells laden mice, **g** measurement of GSH level [105]

Due to low systemic toxicity, biodegradability, changeable mesopore size and substantial loading space for a variety of drugs, hollow mesoporous organosilica nanoparticles (HMON) have attracted more curiosity as an innovative drug delivery mechanism [104]. Huang et al. designed tamoxifen loaded, cupper peroxide nanoparticle (4.4 nm) blocked HMON. To make the carrier pH responsive, HMON was further coated with polyethylene glycol poly(allylamine)-dimethylmaleic anhydride (PEG-PAH-DMMA) as shown in Fig. 4. Acid in the tumor microenvironment reacted with the copper nanoparticle to produce copper ion (Cu^+^) and H_2_O_2_. The Fenton reaction in between H_2_O_2_ and Cu^2+^ generated Cu^+^ and ROS, while one in between H_2_O_2_ and Cu^+^ generated Cu^2+^ and ROS. Cu^2+^ in turn reacted with the GSH to generate GSSG and Cu^+^, elevating the oxidative stress of the cancer cells. Overall, the pH-dependent tumor-responsive HMON had tumor-suppressive effect through generation of ROS storm [105]. Other metals such as calcium also cause cell death by irreversibly switching the calcium signaling to reverse destruction from positive regulation [106].

**Fig. 5:**
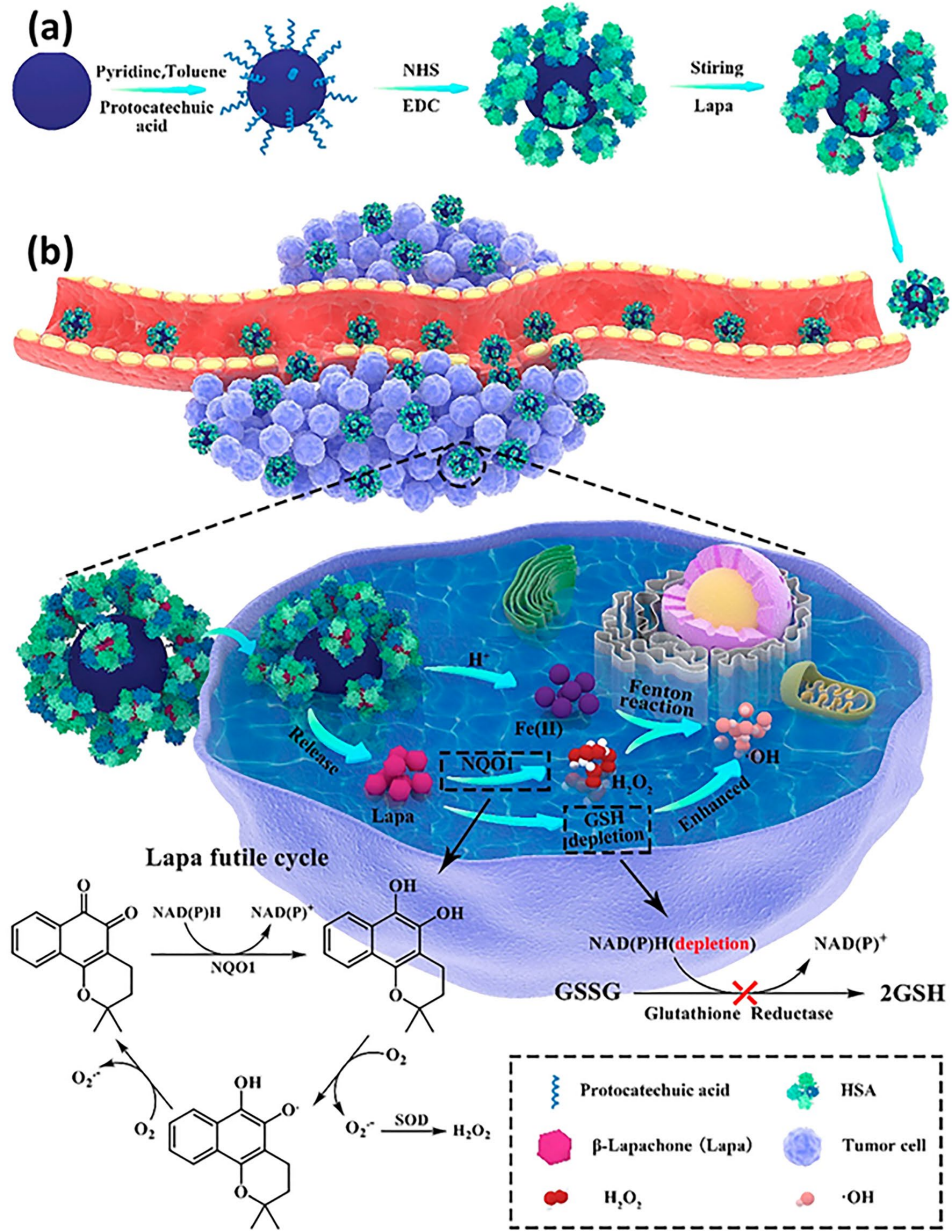
**a** Representation of formation of Lapa-loaded HAS-Fe_2_O_3_ NP. **b** Following intravenous administration, the EPR effect caused HAS-Fe_2_O_3_ NP to concentrate in tumor tissues and be absorbed by tumor cells. Superoxide dismutase (SOD) then transformed the O_2_· into a mass of H_2_O_2_ as a result of the excessive O_2_· produced by the catalytic release of Lapa by the overexpressed NQO1 enzyme. Meanwhile, in the acidic endosomal environment, ferrous ions from self-sacrificing Fe_3_O_4_ combined with accumulating H_2_O_2_ via Fenton reactions to form extremely harmful ·OH leading to cell death. Additionally, consumption of NADPH in the Lapa futile cycle led to GSH depletion, impairing the antioxidant defense system and increasing the sensitivity of tumor cells to •OH produced in the Fe^2+^-mediated Fenton chemical reaction, intensifying the anticancer impact [111]

It is important to note that nanoparticles such as iron oxide nanoparticles possess negligible cytotoxicity at neutral environment, whereas can self-sacrifice to produce Fe^2+^ at acidic pH to produce free radicals demonstrating cancer cell death [107, 108]. Anticancer agents can successfully be delivered via nanoparticles which can enhance the ferroptosis therapy. One of the novel anti-cancer agents, β-Lapachone (Lapa) via futile reaction, produces H_2_O_2_ under influence of quinone oxidoreductase-1 (NQO1) enzyme and nicotinamide adenine dinucleotide phosphate (NADPH). NADPH is utilized as electron donor for inducing futile cycle in between hydroquinone and quinone forms of Lapa depleting intracellular NADPH by 60 mol in 5 min per mole of Lapa. As a cofactor of GSH reductase, NADPH on the other hand keeps GSH in its reduced state. The conversion of GSSG to GSH, which is catalyzed by glutathione reductase, is impeded as a result of the NADPH depletion [109, 110]. In a nutshell, the futile cycle induced by Lapa inhibits the conversion of GSSG to GSH to synergistically elevate tumor’s oxidative stress. Chen and team developed iron oxide nanoparticle (Fe_3_O_4_ NP) connected with protocatechuic acid (PA) via ligand exchange reaction. The human serum albumin (HSA) protein was subsequently covalently linked with Fe_3_O_4_-PA NPs by using 1-Ethyl-3-(3-dimethylaminopropyl) carbodiimide (EDC) to establish amide bonds between the amino groups of HAS and the carboxylic groups in the PA. Finally, Lapa was loaded into the nanoparticle. As shown in Fig. 5, both carrier system and Lapa worked synergistically to induce process of Fenton chemistry by triggering the release of iron ion and elevating H_2_O_2_ level. Lapa, additionally, reduced the amounts of GSH in tumor cells, working in conjunction with extremely harmful ROS to disturb redox homeostasis and increase intratumoral oxidative stress. The protein-modified nanosystem also demonstrated excellent biocompatibility and improved the blood circulation time of Lapa while targeting the specific cancer cells [111].

**Fig. 6:**
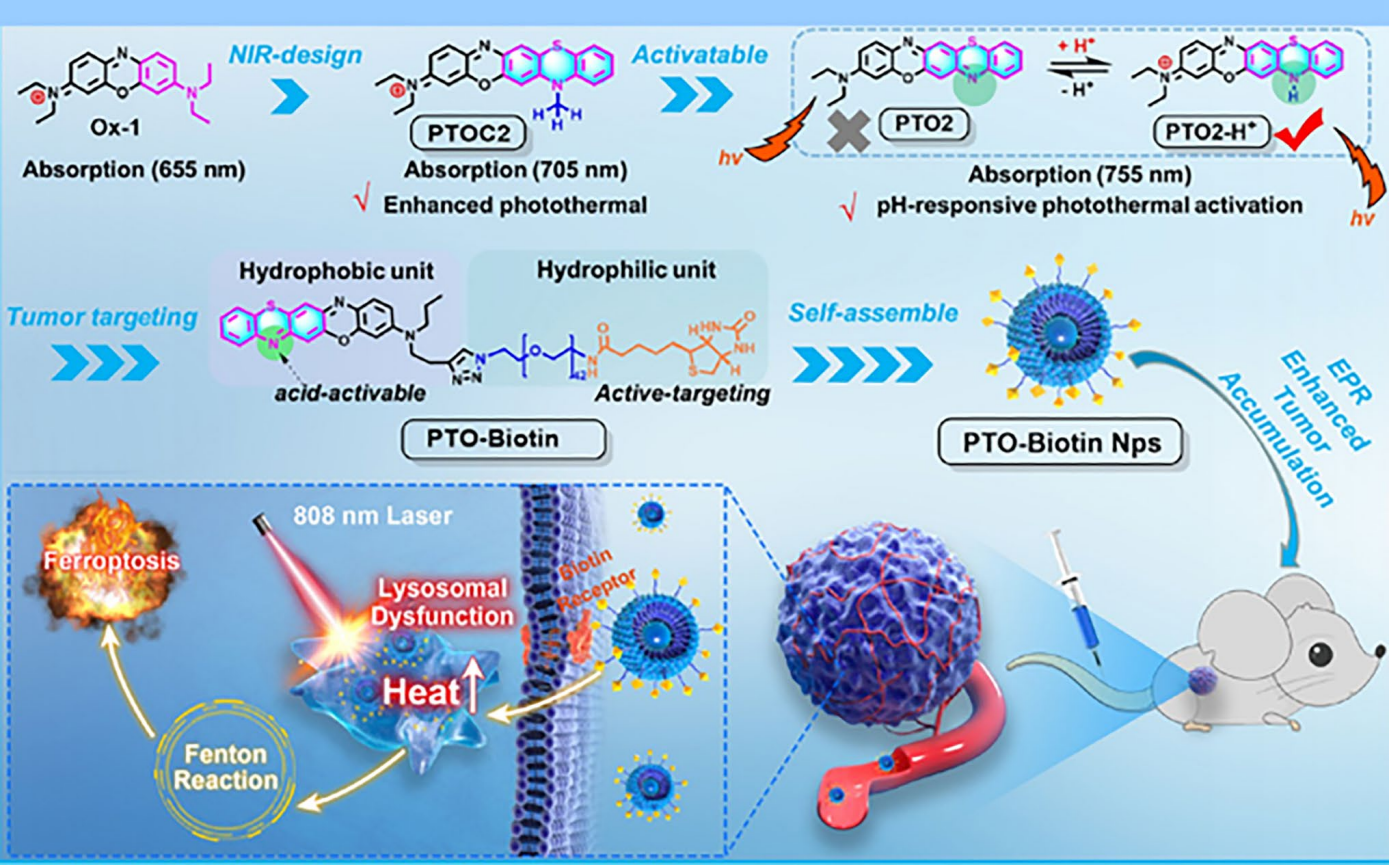
Representation of effect of molecularly engineered pH-responsive photothermal oxazine self-assembled nanoparticles (PTO-Biotin Nps) for spatiotemporal controlled tumor ferroptosis [112]

A spatiotemporal control, near-infrared (NIR) photothermal molecular assembly consisting of phenothiazine-fused oxazine (PTO) biotinylation nanoparticles (PTO-Biotin) (formed by self-assembly of hydrophobic PTO and hydrophilic biotin-PEG chain) showed dysfunctioning of lysosome to promote cell intrinsic Fenton reaction and ferroptosis. The metal-free construction offered a non-intrusive control technique to encourage the Fenton reaction for inducing strong ferroptosis through lysosomal dysfunctions, resulting in compromised autophagy and cytosolic acidification (Fig. 6) [112].

Based on the above studies, modification of nanoparticle surface, ferroptosis inducing agents and GSH depleting substance-loaded nanoparticles would play significant role in clinical translation to solve the problems associated with cancer therapy.

## Challenges of Ferroptosis

Ferroptosis is still awaiting a priority in achieving pre-clinical and clinical settings irrespective of promising applications in cancer treatment. The major treatment barrier for ferroptosis is tumor heterogeneity. Due to variations in the cellular level of iron and gene expression levels associated with ferroptosis, different tumor types and individuals may respond differently to the condition. Consequently, groups who respond to ferroptosis-promoting medicines can be identified using iron levels, iron levels, gene expression and mutations. Interestingly, p53 plays a critical role in the ferroptosis regulation. Xie et al., however, found that tumor cell suppressor (TP53) on wild type of colorectal cancer cells, makes the cells erastin resistant by obstructing the activity of dipeptidyl-peptidase-4 (DPP4) in transcription-independent way, thus blocking ferroptosis [113]. Ferroptosis would not be mediated with high level of GPX4 as seen in stage 3 or 4 thyroid cancer. Knocking down of GPX4 had showed elevation in the iron level and finally oxidation of lipids in FTC133 cells causing the initiation of ferroptosis. Hence, it could be established that under the influence of GPX4 over-expression, effect of ferroptosis would be diminished [114]. Another mechanism that controls ferroptosis and is unrelated to the GPX4/glutathione system is the GCH1-BH4 pathway. Tetrahydrobiopterin production is slowed down by the enzyme GCH1. Lipid peroxidation can be fully eliminated and ferroptosis nearly entirely inhibited by overexpressing GCH1 as observed in melanoma [115]. Concerning pharmacokinetics, ferroptosis-inducing agents possess poor aqueous solubility, limited bioavailability and drug resistance. Erastin could be an excellent example that shows unstable metabolism and poor aqueous solubility leading to unconstrained side effects [116]. Another major challenge is the in vivo application of ferroptosis inducers. For instance, sulfasalazine, sorafenib, cisplatin artemisinin, etc., have been currently considered as promising producers of ferroptosis; however, serious adverse drug reaction, non-specific distribution and high dose limit their application [117, 118].

The treatment should be designed in a way to reach the specific cancer cells without affecting the normal or healthy cells. The ferroptosis inducers, apoptosis inducers or their combination fails to reach the specific or desired site, which in turn, destroys healthy cells. Multi-organ failure or multi-drug resistance, in consequence, decreases the median survival rate.

Nanotechnology could be a promising technique in overcoming aforementioned barriers which due to small size, modifiable structure and targeting ability reduces the dose, improves aqueous solubility and bioavailability and achieves desired targeting ability. Moreover, nanotherapy can combine multiple drugs, photodynamic therapy (PDT) or radiotherapy to improve clinical outcome in cancer patients.

## Unraveling the Scope of Nanoparticulate Systems in Ferrotherapy

To induce ferroptosis, it is important to achieve intracellular accretion of lipid oxygenase and ROS. Therefore, it is encouraging to develop nanosystems, which can suppress GPX4 expression in cancer cells, facilitate or trigger the Fenton reaction and regulate exogenous lipid peroxidation inside tumor cells [119–123]. Catalysis by Fe^2+^ or Fe^3+^ rapidly converts intracellular H_2_O_2_ to highly reactive hydroxyl ions that can trigger oxidative stress. The cancer cells are highly resistant to conventional therapy and apoptosis but are degraded under the impact of ferroptosis, indicating the robustness of ferroptosis in cancer treatment. The concept of drug delivery is not exempted from nanotechnology which involves new scientific discussion and research hypothesis. Nanotherapy has been proposed to promote the accumulation of lipid peroxidases and ROS for three major reasons described above. Lessons from the failure of chemotherapy suggest the extra-ordinary implications of nanosized materials in ameliorating the therapy barriers [124–126].

The section below is associated with the scope of nanoparticles in relation to ferroptosis for cancer therapy. Table 3 briefs about the discussed nanoparticles in inducing ferroptosis-based cancer therapy.

**Table 3:** Illustration of nanoferrotherapeutics involved in treatment of various cancers

Type of nanocarrier system	Chemotherapeutics	Genes	Cancer type	In vitro	In vivo	Inference	References
MOF	Doxorubicin	–	Breast cancer	4T1 cells	Balb/c mice	Doxorubicin-induced ferroptosis causing immunogenic cell death (ICD)	[251]
Gold mesoporousMOF	–	–	Breast cancer	4T1 cells	Balb/c mice	Cu and Fe ion bridged using disulfide bond induces ferroptosis by depleting GSH, finally inhibiting GPX-4 level	[137]
MOF	Doxorubicin	–	Breast cancer	4T1 and MCF-7	Mice (name not mentioned)	The drug-loaded nanoparticle showed great potency and clinical translation by targeting CD44 over-expressed cells	[141]
Fe(III)– porphyrin-MOF	Oxaliplatin	–	–	Breast cancer	4T1 cells	The therapy increased the level of H_2_O_2_ and (IFN-γ), which in turn caused ferroptosis of cells	[145]
Fe-MOF	–	–		Breast cancer	4T1 cells	Aptamer PD-L1-attached glucose oxidase and PEG-modified iron-based MOF were an innovative strategy to disrupt iron homeostasis and hinder intracellular redox	[21]
RBC membrane camouflaged MOF	Doxorubicin	–	Breast cancer	MCF-7	Balb/c nude mice	Results illustrated that self-assembled RBC membrane camouflaged MOF could amplify the oxidative stress of ROS, reduce glutathione potentiating remarkable anticancer effects	[146]
MOF	Doxorubicin	–	Breast cancer	4T1 cells	Balb/c mice	The nanoplatform downregulated GPX4 to induce ferroptosis	[251]
Nanophoto-sensitizer	–	–	Human melanoma cancer	A375	Nu/Nu female mice	A photodynamic therapy-based nanosheets of graphitic carbon nitride functionalized with mitochondria-targeting iridium (III) polypyridine complexes caused hypoxic environment resulting in cell death	[225]
Theranostic nanoparticle composed of iron ions, cinnamaldehyde prodrug and amphiphilic polymer skeletal (FCS/GCS)	–	–	Breast cancer	4T1 cells	Balb/c mice	The preparation with the help of CA induces Fenton reaction, generated ·OH and accelerated lipid peroxides (LPO) accumulation and accordingly augments ferroptosis	[229]
Bonsai-inspired AIE nanohybrid photosensitizer	–	–	Colon cancer	MC38 cells	Balb/c	The Bonsa-inspired nanopreparation in presence of white light irradiation produced hydroxyl radical and depleted GSH to induce ferroptosis in cancer cells	[240]
Cell membrane decorated iron-siRNA nanohybrid	–	Anti- SLC7A11 siRNA	Human oral squamous cell carcinoma	CAL-27	Male Balb/c	The nanohybrid system elevated the ROS level to show synergic anti-cancer effect in vivo	[96]
Magnetic lipid nanoparticle	–	siDECR1	Castration-resistant prostate cancer	C4–2B or C4-2BEnz cells	Nude mice (name not mentioned)	The biomimetic nanoparticles were stable, safe and effective in showing remarked inhibition of distant organ metastasis	[218]
Graphene oxide-PEG-PEI nanoparticle	Sorafenib	PD-L1 siRNA	Hepatocellular carcinoma	MHCC97H cells	C57BL/6 mice	The developed preparation reduced the expression of GPX4 in the intrahepatic tumor regions in immunocompetent mice	[219]
Liposome	Artemisinin	–	Lung carcinoma	LLC cells	Balb/c nude mice	The remarkable autophagy-mediated ferroptosis-involved cancer-therapeutic efficacy is suggested by therapeutic outcomes both in vitro and in vivo which is further confirmed by transcriptome sequencing	[160]
Nanostructured lipid carrier	Doxorubicin, ferrocene	–	Breast cancer	4T1 cells	Balb/c mice	TGF-β receptor inhibitor along with ferrocene and doxorubicin-loaded NLC inhibited mammary cancer metastasis by extracellular as well as intracellular hybrid mechanism	[164]
Liposomes	–	–	Breast cancer	4T1 cells	Balb/c mice	An amalgamation of sonosensitizing agent (PpIX) and ferumoxytol in liposomes induced apoptosis and ferroptosis overcoming the tumor resistance	[215]
Iron oxide nanoparticles	Paclitaxel	–	Glioblastoma	U251 and HMC3 cells	Balb/c-nu mice	PTX-IONP reduced the ability of cells to invade and migrate, elevated ROS, iron ions and lipid peroxidation, enhanced the expression of the autophagy-related proteins LC3II, and Beclin1 and suppressed the expression of the p62 and GPX4	[173]
Nanozyme	Cisplatin	–	Ovarian cancer	SKOV3/DDP cells	BALB/cJGpt-Foxn1nu/Gpt mice	The formulation was able to induce both apoptosis and ferroptosis with the help of ultrasound treatment for cisplatin-resistant cancer cells	[193]
Nanozyme	–	–	Triple-negative breast cancer	MDA-MB-231 cells	Balb/c nude mice	The imaging-based nanosystem used SPIO and Avastin to induce tumor starvation and ferroptosis	[263]
Nanozyme	–	–	Breast cancer	4T1 and MCF-7 cells	Balb/c mice	Tumor ablation was accomplished with Fenton reaction-independent ferroptosis driven by photothermal nanozyme	[197]
Nanozyme	Gemcitabine	–	Pancreatic cancer	PANC02	Balb/c mice	GEM and MnFe_2_O_4_ can synergistically improve anti-cancer profile via ferroptosis and GEM-mediated chemotherapy	[198]
Human serum albumin nanoparticle (HAS-NP)	Pt (IV)	–	Ovarian cancer	SKOV3	Mice (name not mentioned)	The prodrug of platinum in HAS nanoparticles was effective for ovarian cancer cell resistance to platinum	[241]
Iron-doped calcium carbonate nanoparticles	Pt(IV)	–	Breast cancer	4T1 and CT26 cells	Balb/c	Iron-doped platinum-SA-based CaCO_3_ showed development of ROS and lipid peroxidation to mediate cancer cell death	[242]
Zeolite imidazolate framework (Zif-8)	–	–	Head and neck squamous cell carcinoma	HN6	Male Balb/c	DHA and SNP enveloped nanoreactor system prompted Fe^2+^ and NO release to initiate ferroptosis and apoptosis synergistically	[247]
Hypoxia-responsive nanoelicitor	Mitoxantrone	–	Colorectal cancer	CT26 cells	Balb/c nude mice	The simultaneous co-stimulating effects of CA and MIT resulted in an elevated antiproliferative and anti-cancer immunity, which in turn aborted the system Xc^−^ to GPX4 pathway and increased the iron-initiated tumor cell destruction	[248]

### Metal–Organic Framework Nanoparticles

Metal organic framework (MOF) nanoparticles (MOF-NP) belong to the class of coordination compounds employing clusters of metal ions or organic ligands wherein the development occurs through an amalgamation between most of metals, central atom and the ligand [127]. These peculiar nanosystems garnered interest owing to their remarkable properties, including hydrothermal, thermal, mechanical and chemical stability, specifically applicable for industrial use [128]. High volumetric absorption capacity, efficient drug loading capacity, huge surface area and catalytic potential signify these materials for advancing clinical benefits. Not confining to this, the porous features with adjustable and ensembled cavity enabled their biomedical applications. In the biosensing and molecular imaging field, these materials exhibited distinguishing parameters ranging from biocompatibility, biodegradability and water stability to showing binding or conjugating capability with organic blocks or metals [129, 130]. Further interaction with functional groups or specific functionalized molecules extends their role in targeting cells or organs. Stimuli-responsive MOFs could deliver bioactive molecules such as bioimaging agents, photosensitizers, and fluorescents for chemotherapy, therapeutic agents, and PDT. MOF-NP meticulously shows considerable attributes such as structural feasibility, enormous surface area, and modifiable chemical properties with excellent porosity to meet desired therapeutic, diagnostic, and theranostic demands [131].

**Fig. 7:**
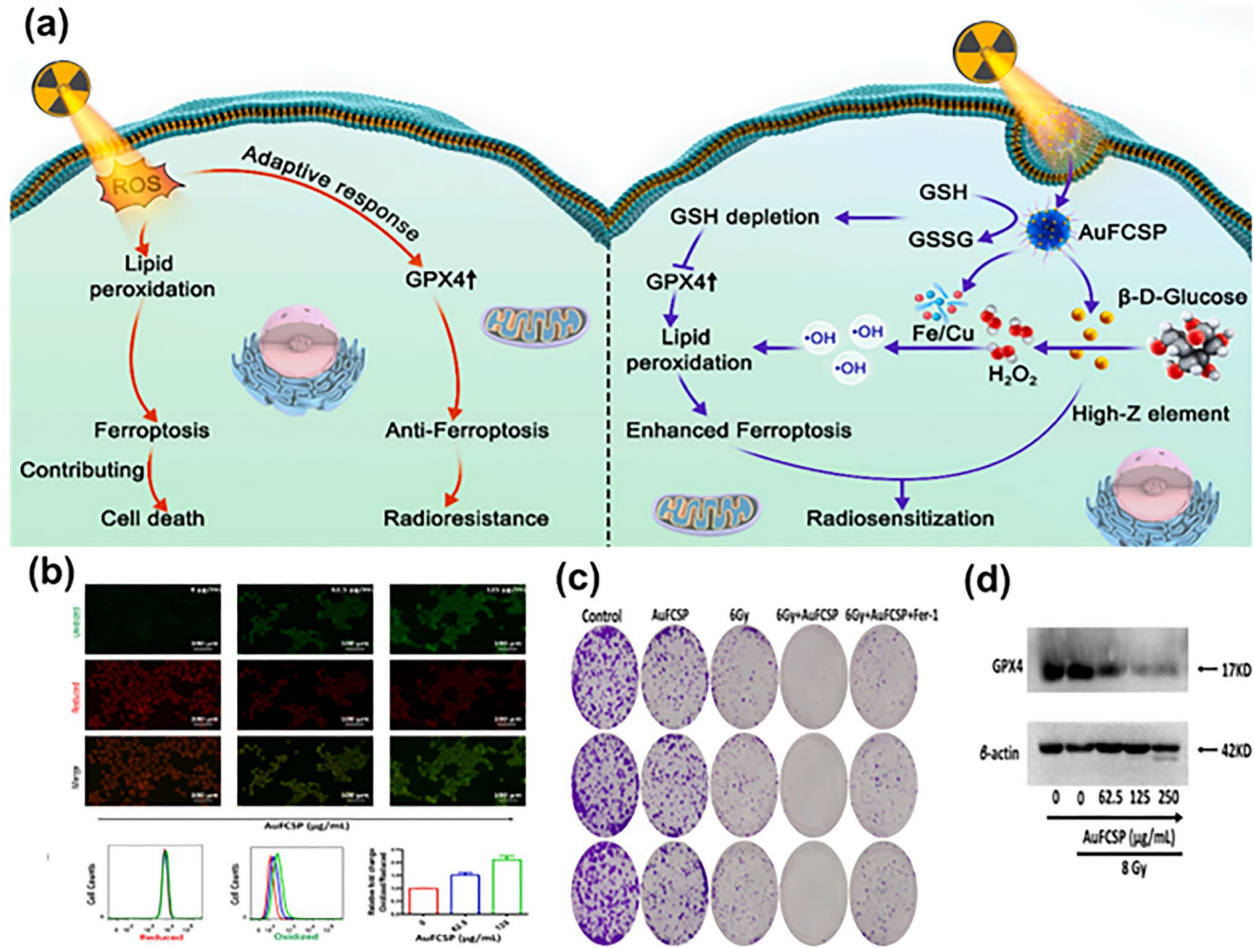
**a** Schematic illustration of synergistic effect of radiotherapy and ferroptosis mediated by Au-FCP-MOF-NP. The left side represents the negative role of GPX4 stimulated by radiotherapy to resist ferroptosis and finally radio-resistance, while the right side shows induction of ferroptosis due to intratumoral release of Fe-Cu dual ion along with Au NP which catalyzed β-D-glucose oxidation to develop gluconic acid. Increased amount of H_2_O_2_ released toxic hydroxyl radicals which induced ferroptosis. Overall, radiosensitization increased due to synergic effect of high Z element (Au) and ferroptosis; **b** fluorescence images of cell (4T1) treated with Au-FCP-MOF-NP to evaluate peroxidation with respective flow cytometry analysis; **c** Au-FCP-MOF-NP and IR treated 4T1 cells showing extend of colon formation and **d** western blotting results showing expression of GPX4 with various concentration of Au-FCP-MOF-NP with or without exposure of IR. Reproduced with permission from Ref. [137]

A continuous breakthrough in cancer therapy led scientists to evaluate anti-proliferation results based on agents showing synergistic roles. Radiotherapy is a well-known cost-effective cancer treatment that uses ionizing radiation to break the double-strand DNA or generate ROS or high-energy ionizing radiation (IR) to damage lipids, proteins, and nucleic acids causing cell death through multiple modes (autophagy, necrosis, apoptosis, and mitotic catastrophe). The downfall observed using such an approach is due to radioresistance. This creates a necessity and pressurizes the personnel working in the field of cancer therapy to develop anti-radioresistant strategies for improving the quality of life. Outstandingly, ferroptosis has been discovered to be involved in IR progression. Studies claimed that IR induces ferroptosis together with necrosis and apoptosis to end cell fate [132–134]. The disease-free survival time and therapeutic potential of IR also amended in cancer patients after a positive surge in the level of ferroptosis. The underlying cause behind such a positive response could be the ferroptosis inducers that tweaked the effect of radiotherapy [135, 136]. After understanding the relation between ferroptosis and radiotherapy, Liang and team developed a tumor microenvironment-responsive MOF having an amalgamation of Cu and Fe dual ions bridged using pegylated disulfide bond (FCP-MOF) applied as ferroptosis inducer that instigated ferroptosis by synergistic GPX4 inactivation and Fenton/Fenton-like reaction. FCP-MOF works by the accretion of lipid hydroperoxides based on two different aspects: one is by GSH depletion causing GPX4 inactivation helping in reducing the scavenging of ROS and lipid peroxides, while the second is a Fenton/Fenton-like reaction which extends ROS production. Since cancer cells abundantly require glucose to proliferate, applying natural glucose oxidase (Gox) in the tumor microenvironment could augment the H_2_O_2_ concentration causing cell lysis by cancer starving therapy. But, poor stability, high cost of purification and synthesis and changeable biological environment impede its biomedical application. As gold nanoparticles (Au NP) could mimic the Gox property, scientists had grown the Au NP in situ n FCP-MOF (Au-FCP-MOF-NP) to convert the over-uptake glucose into gluconic acid showing a cascade of H_2_O_2_ and Fenton/Fenton-like reaction **(**Fig. 7**)**. To verify the hypothesis, the level of ROS production and GPX4 depletion was evaluated. As expected, Au-FCP-MOF-NP inactivated GPX4 protein expression by depleting the GSH level leading to the onset of ferroptosis. Lipid peroxidation rate was assessed in 4T1 cells showing green fluorescence due to increased lipid oxidation, while no change in the red fluorescence of abridged lipids was observed. Within tumor microenvironment, the depletion of GSH was prompted by the disulfide-thiol exchange reaction. The results were further confirmed by flow cytometry indicating lipid peroxidation by Au-FCP-MOF-NP. The radiosensitizing effect of Au-FCP-MOF-NP was also determined through a colon formation study, wherein the developed preparation significantly impeded the colon formation under the effect of IR indicating their sensitizing ability. The biosafety results by hemolysis and the histopathological study confirmed the biocompatibility of the preparation. Thus, a high-Z element enhancing and ferroptosis-inducing synergistic effect liberated significant anti-tumor response both in vitro and in vivo [137].

**Fig. 8:**
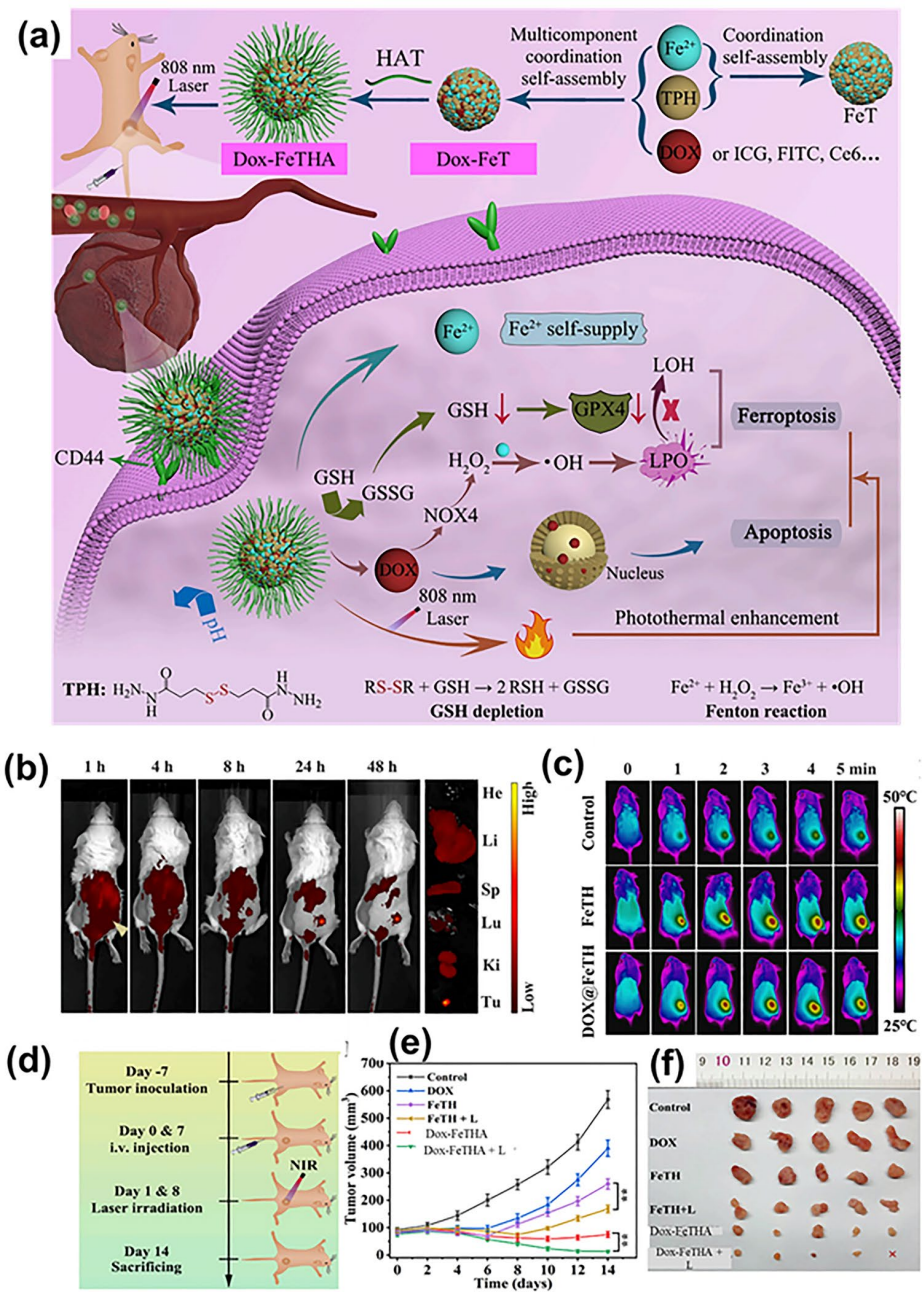
**a** Schematic representation of development process of Dox-FeTHA-aMOF with its application in photothermal-assisted synergistic ferroptosis-apoptosis cancer therapy; **b** time-dependent fluorescence descriptions of 4T1 tumor-bearing mouse post-injection with dye (Cy5.5) labeled aMOF with detected fluorescence in major organs at 24 h post-treatment; **c** infrared thermal images under 808-nm laser irradiation; **d** representation of development of tumor model and respective treatment strategy; **e** illustration of tumor volume with **f** digital pictures of excised tumor lesions. Reproduced with permission from Ref. [141]

The success of chemotherapy to suppress tumor is measured by the extent of caspase-dependent apoptotic cell death, but the reality is far from satisfactory results due to heterogeneity or acquired resistant to apoptotic therapy [138]. Ferroptosis, in comparison with apoptosis, is not governed by apoptosis-associated factors, thereby bypassing apoptosis embargo [139, 140]. Fe^2+^ is a resourceful element that exhibits a fascinating role in ferroptosis execution; (i) Fe^2+^ augment intracellular ROS propagating lipid peroxidation; (ii) it regulates mitochondrial electron transport complexes and ROS-leveraging substances; (iii) Fe^2+^ escalates lipid autoxidation or PUFA peroxidation. Various pioneer research has shown the explicit role of Fe^2+^ in cancer therapy using nanoplatforms. A study reported all active amorphous MOF (aMOF) for photothermal-assisted synergistic ferroptosis-apoptosis cancer therapy. The system was generated by the one-pot co-assembly of Fe^2+^, doxorubicin (Dox) and 3,3′-dithiobis(propionohydrazide) (TPH), further modified with TPH-functionalized hyaluronic acid (Dox-FeTHA). Dox-FeTHA, being an amorphous structure, displays GSH, pH and laser triple-responsive degradation aspects. Dynamic light scattering results demonstrated successful developed nanopreparation having size and zeta potential of 122.4 nm and − 32.3 mV, while the transmission electron microscopic (TEM) images showed monodispersed spherical nanoparticles. The in vitro cellular uptake studies showed a high uptake of Dox-FeTHA-MOF in CD44 overexpressed cells as compared to medium expressed cells indicating receptor-mediated endocytosis. Inductively coupled plasma-optical emission spectrometry (ICP-OES) technique showed an increase in iron content with increase in incubation time, which further inclined after laser irradiation. An abundance of disulfide bond in Dox-FeTHA and FeTH leads to enormous consumption of GSH, making the former more potent. The cell counting kit 8 (CCK-8) assay was used to investigate the therapeutic effect of preparation in two positive cells line (4T1 and MCF-7) and in a normal cell line (L929 cells). The toxicity of Dox-FeTHA was much higher in 4T1 cells as compared to MCF-7 and normal cell. It was also observed that individual Dox-FeTHA aMOF had slight cytotoxicity at iron concentration less than or equal to 6.4 μg mL^−1^, suggesting satisfactory cytocompatibility. After 808-nm laser irradiation, the viability of cells remarkably reduced showing the intracellular photothermal effect of Dox-FeTHA-aMOF. Animals treated with Dox showed reduction in body weight owing to the toxicity; however, other group manifested regulated body weight (Fig. 8) [141]. Overall, the synergistic approach of using such nanomedicine can have great clinical translation potential.

**Fig. 9:**
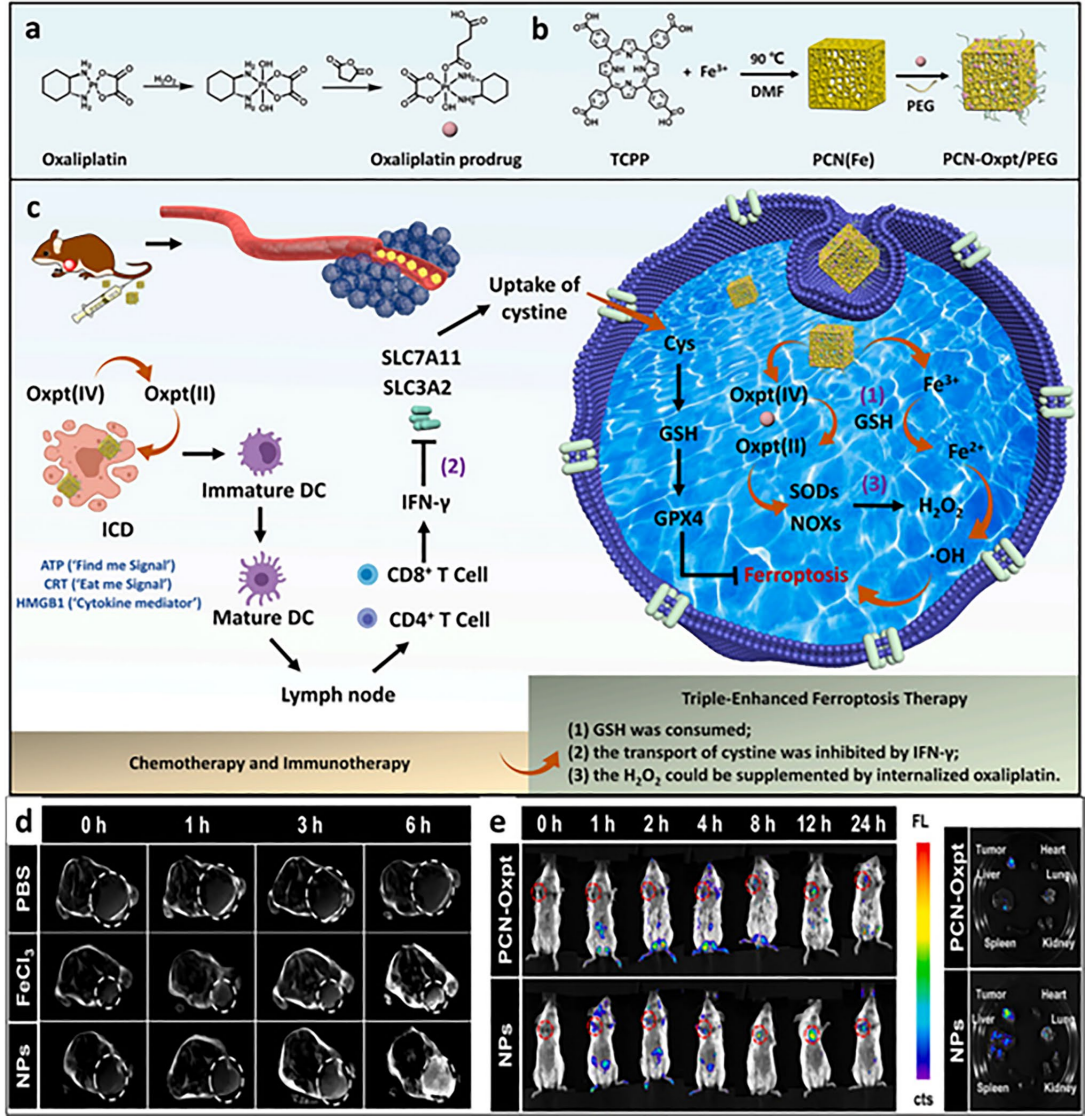
The triple-hit effect of (PCN-Oxpt/PEG) for inducing chemotherapy, ferroptosis and immunotherapy, **a** representation of development of oxaliplatin prodrug and **b** PCN-Oxpt/PEG, **c** magnetic resonance images of mice after treatment with PBS, iron chloride and nanoparticles showing accumulation at tumor site [145]

**Fig. 10:**
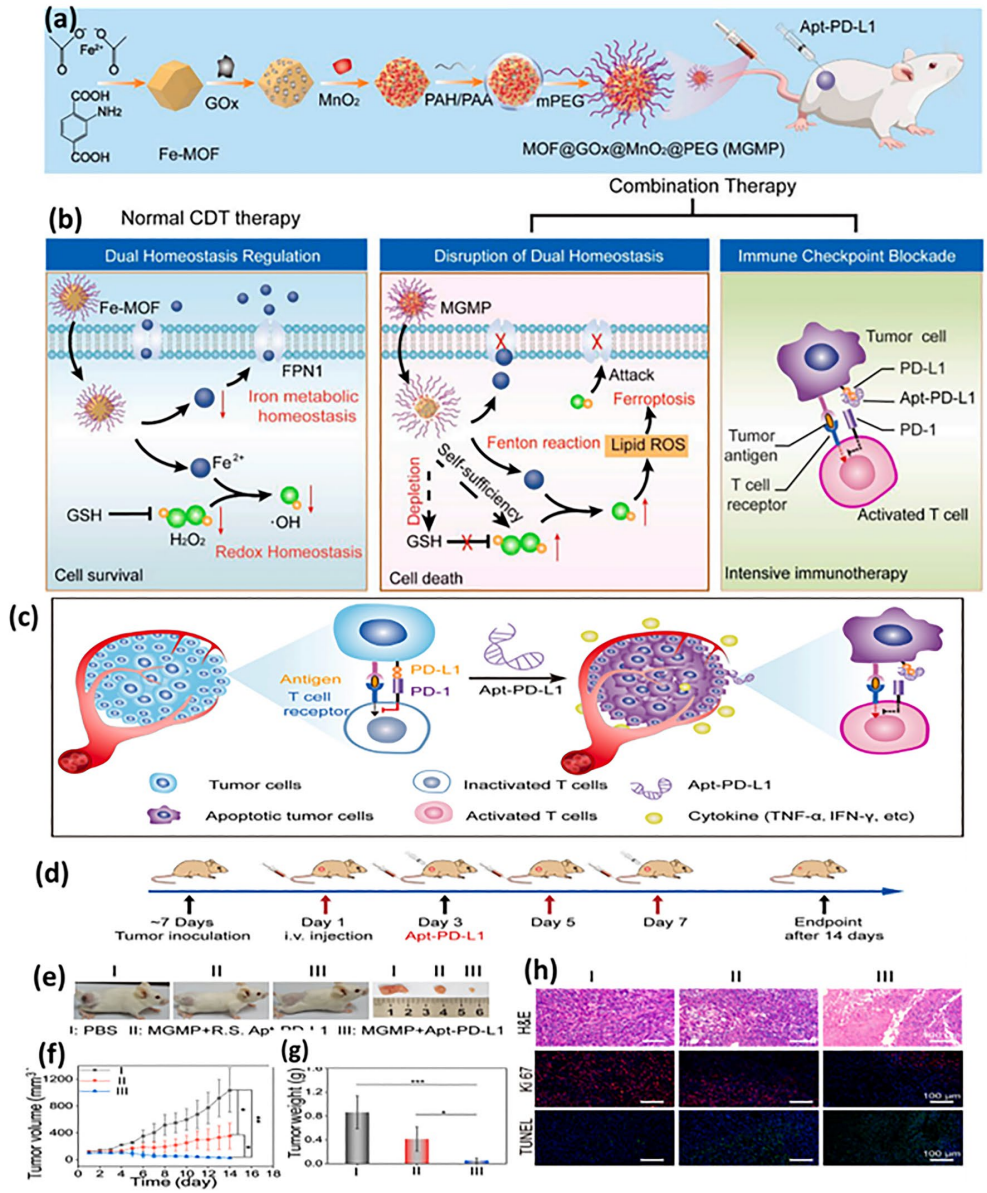
**a** Schematic representation of development of PD-L1-targeted aptamer-grafted iron-based MOF, **b** the developed therapy based on ferroptosis-mediated immunotherapy disrupts iron homeostasis and redox balance through inactivation of GSH, ROS accumulation and transferrin 1 downregulation to finally suppress PD-L1 checkpoints, **c** illustration of blocking the targeted site (PD-L1), **d** illustration of therapy-induced in mouse models, **e** digital images of different treatment groups, **f** estimation of tumor volume (mm3) and **g** tumor weight of xenograft model of mouse bearing 4T1 cells, **h** tumor section analysis through H&E-stained images, analysis of Ki 67 and TUNEL test. Reproduced with permission from Ref. [21]

Fe ions convert the intracellular H_2_O_2_ into hydroxy radicals which in turn aggregate the lipid peroxidation and disrupt the redox balance of cells causing their lysis. This ground breaking strategy highlighted the use of various iron-based nanoparticles in ferroptosis therapy [142–144]. A nanotheranostic was developed in a study using modified oxaliplatin prodrug and poly (ethylene) glycol (PEG) on Fe(III)-porphyrin metal–organic frameworks (PCN-Oxpt/PEG), wherein Fe(III)-porphyrin MOFs possessed magnetic resonance imaging (MRI) and fluorescence potential, while the cloak of PEG improved the biological half-life. The developed preparation showed ferroptosis in multiple ways: (i) the prodrug consumed GSH, inhibiting the activation of GPX4; (ii) oxaliplatin triggered CD8^+^ T cells and produced IFN-γ limiting GPX4 activation; (iii) after internalization, oxaliplatin prompted the generation of H_2_O_2_ generation by activating NADPH oxidases (NOXs) and superoxide dimustase-mediated superoxide anion (O_2_^⋅−^) dismutation causing lipid peroxidation. The triple hit effect of chemotherapy, ferroptosis and immunotherapy was observed in 4T1 bearing tumor mice showing advanced inhibitory effect as compared to single therapy. Conclusively, the multi-functional nanoplatform with image guiding and tumor-responsive therapy is excellent platform for overcoming pitfalls of conventional therapy (Fig. 9) [145]. Iron metabolism homeostasis and redox homeostasis regulate the level of iron and ROS against several changes in cancerous cells, limiting cancer cell ferroptotic effect. A disruption in iron ions and ROS homeostasis can be useful in elevating ferroptosis-based therapeutic efficiency. Ferroptonin-1 (FT-1), an iron-regulated transporter, plays significant role in the regulation of iron homeostasis that expels out the unexploited iron from the cell to bypass large amount of iron retention. Zhang and team accomplished an PD-1 aptamer-mediated targeted therapy which acted as homeostasis disruptor. Polyethylene glycol (PEG), glucose oxidase (Gox) and MnO_2_ were grafted on iron-based MOF using a hydrothermal method. The fascinating features of MnO_2_ were used to downregulate the GSH level which then disrupts the redox homeostasis. Gox facilitates glucose oxidation to produce hydrogen peroxide, while PEG extends the system’s biocompatibility. Here, iron-based MOF not only acted as carrier system but also encouraged development of hydroxy ion via Fenton chemistry for chemodynamic therapy. Moreover, the ELISA results suggested that the developed nanopreparation reduced the expression of FT-1 due to its strong potency to generate ROS and hydroxyl ion causing ferroptosis of cancer cells. The targeted preparation promoted the infiltration of immune cells inducing both ferroptosis and apoptosis of breast cancer cells (Fig. 10) [21]. The release of oxaliplatin and iron ions from nanoparticle explicitly generated hydroxyl ion in abundant amount by Fenton reaction leading to lipid peroxidation and finally ferroptosis. Overall, it was established that multifunctional nanoplatform with porphyrin metal–organic frameworks, Fe(II) and PEG not only helps to attain anti-cancer effect but also detect the cancer cells.

The strategy of delivering the “perfect” MOF relies upon its deliberate and sophisticated ligand envelope that attracted intensifying recognition in drug delivery. However, defect engineering is also a revolutionizing approach for catalysis, tuning sorption and modifying physical characteristics. One of the major hurdles observed nowadays is the unsynchronized dispersion of medicinal agents in MOF nanoplatforms, which inspires the scientists to work in the direction to develop a set of applications as tandem catalysts. In a study, three-target one-approach-based nanosystem (MOF) was constructed to deliver a ferroptosis-inducing agent, levitate ROS level with simultaneous downregulation of GSH level. The so-called defect structure allowed the Fe ion to link with the MOF through a 1,4-benzenedicarboxylate linker ligand and decorated with biomimetic erythrocyte membrane to overcome drug-resistant malignancy. The tumor microenvironment destroyed the camouflaged system liberation Fe^3+^ and Pseudolaric acid B (PAB) (natural diterpenoid responsible for angiogenesis, metastasis arrest and sensitivity enhancement of neoplastic cells toward radiotherapy). Damage of oxidative phospholipids triggers ferroptosis, thus controlling the permeability and fluidity of the cell membrane to suppress the expression of P-gp protein to overcome drug resistance.

The western blot technique was used to evaluate the level of GSH and thereby GPX4. The PMNP-DOX@RBC liberated Fe^3+^ and depleted GSH via reduced reaction causing release of glutathione (GSSG) and ferrous ion (Fe^2+^). The following reaction entails the mechanism.

$${\text{2Fe}}^{{{3} + }} + {\text{2GSH}} \to {\text{2Fe}}^{{{2} + }} + {\text{GSSG}} + {\text{2H}}^{ + }$$

Animals treated with PMNP-DOX had 82% antitumor rate as detected by tumor weight measurement; contrastingly, those treated with PMNP-DOX@RBC had 87% anti-tumor rate.

Hence, using a complimentary ferroptosis-apoptosis mechanism, the MOF-membrane disguised nanoplatform may consume GSH, exacerbate ROS oxidative stress and finally kill tumor cells confirming them as potential treatment option in oncotherapy [146].

### Lipid Nanoparticles

Lipid nanoparticles (LNPs) are nanocarriers in size range of 100 nm that are derived from a variety of lipids and other biochemical compounds [147–155]. LNPs have the ability to dissolve biological barriers (biobarriers), which enables them to preferentially assemble outside of disease-target cells once more for effective results. The majority of chemically and physiologically unstable substances employed in pharmaceuticals were insoluble in aqueous solutions [156, 157]. The lipid-based nanoparticles (LBNPs) technology is one of the most promising drug carriers for bioactive chemical molecules. By enhancing anticancer effects of various chemotherapeutics, its current use in chemotherapy has changed the way of cancer therapy. LBNPs have excellent temporal and thermal stability, ease of preparation, high load potential, high manufacturing output and low manufacturing costs as they are made from naturally existing sources [158, 159].

Li and the group constructed artemisinin (ART) and CuO_2_ nanodots (CPNs) co-loaded liposomes as ferroptosis and autophagy-inducing cancer nanotherapy. Fe-based catalyst has sparked tremendous attention as they can arrest the overexpressed H_2_O_2_ while triggering action at mild acidic condition of tumor microenvironment. Meanwhile, the rigorous catalytic conditions (pH 2–4) and restricted endogenous H_2_O_2_ level of the Fenton reaction still prevent the achievement of a substantial and adequate potency. To address the low H_2_O_2_ level in the tumor environment, the component of CPNs was first made using a simple peroxidation reaction, which may operate as a self-supplying Fenton agent. H_2_O_2_ and Cu^2+^ were concurrently liberated from CPNs in response to a mildly acidic TME, followed by an immediate Cu-based Fenton-like catalytic reaction. Additionally, it was determined that Cu^2+^ may significantly increase the amount of ROS radicals produced by breaking the artemisinin’s endoperoxide bridge. To co-deliver ART and CPNs, a composite liposomal nanosystem called Lipo-ART@CPNs was designed. Following incubation with LipoART@CPNs in conjunction with ultrasound irradiation, around 77% of tumor cells were destroyed, showing the stronger therapeutic potential, which was further verified by Calcein-AM/PI staining. Additionally, the autophagy inhibitor CQ was developed to assess the impact of ART-related autophagy as autophagy may be crucial for the survivability of tumor cells that have been treatment with Lipo ART@CPNs. As a consequence, CQ interfered with the Lipo-ART@CPNs-treated lowered cell viability, demonstrating that the autophagy reaction effectively promoted the demise of LLC cells. Flow cytometric analysis served as additional confirmation of the findings. Transcriptome sequencing further supported the remarkable autophagy-enhanced ferroptosis-involved associated cancer-therapeutic efficacy, confirmed by both in vitro as well as in vivo studies. Conclusively, with the incorporation of CPN potentiated the release of Cu^2+^ and H_2_O_2_ under the acidic microenvironment, while Cu-dependent Fenton reaction generated deleterious ·OH radical (catalytic reaction I). Moreover, to liberate the ROS radicals, Cu^2+^ fragmented ART’s endoperoxide bridge (catalytic reaction II). The therapy was further extended by irradiating with ultrasonic radiation to trigger adequate release of drug and improve catalytic activity, suggesting it an instructive nanoferrotherapy in the area of cancer catalytic medicine [160].

Nanoparticles, particularly nanostructured lipid carriers (NLC), have shown a global shift. They possess tremendous advantages, including enhanced cutaneous hydration, improved therapeutic impact, higher stability of the encapsulated active components, longer shelf life and worldwide consumer acceptance [161]. To create NLC, a combination of solid and liquid lipids and a surfactant or co-surfactants are used. Argan oil, grape seed oil and pumpkin oil are just a few of the oils that have already been utilized in the construction of NLC. NLCs development involves the use of emulsifiers, liquid and solid lipids, and suitable biodegradable lipids. Incorporating liquid lipids (oil) tends to cause structural flaws in solid lipids causing unsymmetric crystalline arrangement which inhibits drug leakage and provides a substantial drug load. Interest in NLCs has increased recently as a potential replacement for SLNs, emulsions, polymeric nanoparticles, liposomes, microparticles, etc. These nanocarriers can be used to deliver medications that are both lipophilic and hydrophilic. For the administration of medications via ophthalmic, oral, topical, parenteral, pulmonary and transdermal routes, NLCs have emerged as a viable carrier system [162, 163].

**Fig. 11:**
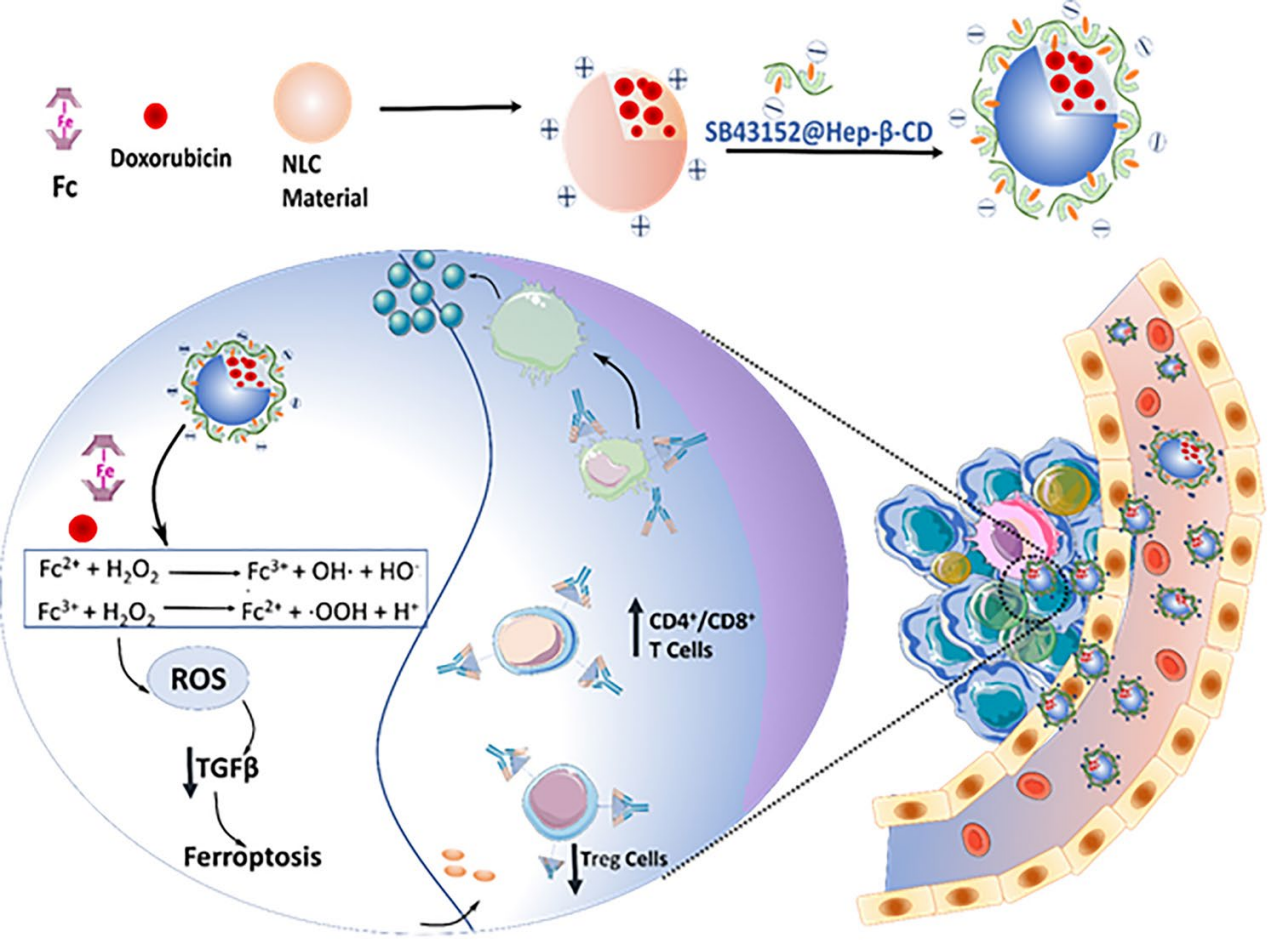
Representation of ferrocene (Fc) and Dox-loaded cationic NLCs developed by emulsion solvent evaporation method, which was then decorated with β-cyclodextrins grafted heparin (β-CD-HEP) by the help of electrostatic interaction. The preparation triggered Fenton reaction, reduced TGF-β secretion and induced immune responses for ferroptosis-mediated anti-cancer therapy

It was reported that ferric (Fe^3+)^ and ferrous (Fe^2+^) convert H_2_O_2_ to ·OH via Fenton reaction. Comparatively, ferrous ion has significantly higher catalytic potential that ferric ion by a range of magnitude. The problem encountered is that the Fe^2+^ is present in low concentration in cellular pool, while numerous iron binds with the ferritin. Chemotherapeutic agents such as doxorubicin (Dox) can also be demarcated as ferroptosis inducers due to its ability to cause ROS-mediated cell death or immunogenic cell death (ICD). In a study, ferrocene (Fc) and Dox-loaded cationic NLCs were developed by emulsion solvent evaporation method, which was then decorated with β-cyclodextrins grafted heparin (β-CD-HEP) by the help of electrostatic interaction. TGF-β receptor inhibitor known as SB431542 was placed inside the cavity of β-CD-HEP. The developed nanoparticle showed a particle size of 124.80 ± 3.79 nm and a zeta potential of − 11.74 ± 1.77 mV, which is due to the electrostatic interaction between β-CD-HEP and NLCs. Fc was used in this work to convert H_2_O_2_ to ·OH. The Fenton reactions by product, ·OH, are a type of extremely toxic ROS which triggers lipid peroxidation commencing the ferroptosis pathway. Therefore, DOX and Fc stand to reason as tandem to elicit ferroptosis. The expression of cytochrome C was well demonstrated by a western blot study after treatment with the heparinized NLC, which confirms mitochondrial damage and induction of apoptosis. Moreover, the expression of MMP-9 was also reduced significantly. The preparation demonstrated modulation in the tumor microenvironment by decreasing the expression of tumor-associated fibroblasts (TAFs) marker, α-SMA along with blocking the TGF-β pathway. As a result, the nanoparticles significantly enhanced the treatment effect of DOX, not only in anti-metastasis and tumor inhibition rate (TIR) but also in modulation of the tumor microenvironment under the influence of Fenton reaction (Fig. 11) [164].

### Iron Nanoparticle

The significance of ferroptosis in combination with nanoparticles for cancer therapy is becoming more apparent as a result of the development of nanomaterials [26, 165–169]. Induction of ROS generation and lipid peroxidation are the major underlying cause for ferroptosis. The emerging iron-based nanosystems work under such mechanism by promoting Fenton reaction, inhibit GPX4 expression and regulate cell peroxidation exogenously [170, 171]. The nanosystems are manufactured in a way to respond toward tumor microenvironment, liberating species for promoting regulated cell death. Currently, numerous iron-based nanoparticles have been approved by US FDA specifically iron oxide nanoparticle having hydrodynamic size less than 50 nm. Nanoparticles can be passively targeted to cancer cells through the improved permeability and retention effect in tumor tissues after being entered into blood arteries while being rejected as foreign substances by mononuclear macrophages [172]. Chen et al. developed paclitaxel-loaded iron-oxide nanoparticle (PTX-IONP) having size and zeta potential of 36.18 nm and − 29 mV, respectively. The drug loading was further confirmed by UV–Vis spectrophotometer and high-performance liquid chromatography (HPLC) method confirming successful loading of drug. Inhibitory results of PTX and PTX-IONP were assessed on U251 cell line and human microglia clone 3 (HMC3) cells, wherein both PTX and PTX-IONP suppressed the U251 cell without showing any significant difference, while on HMC3 cells, PTX showed improved inhibition than PTX-IONP indicating a lesser effect on HMC3 cells. However, the next investigation using PTX-IONP, PTX, PTX-IONP + 3MA and PTX-IONP + rapamycin on U251 cells showed remarked cell death with inhibition on invasion and migration effect with PTX-IONP as compared to PTX alone. It is interesting to note that the autophagy inhibitor 3-MA reduced PTX-IONP’s inhibitory effect, while the autophagy enhancer rapamycin could increase it. Additionally, in the PTX-IONP group compared to the PTX group, the relative expression of the autophagy-related proteins LC3II to LC3I and Beclin1 increased, while the relative expression of p62 dropped. The effect of PTX-IONP may also be inhibited by 3-MA and strengthened by rapamycin. Since chemotherapeutics had pronounced toxic effects which limit their therapeutic efficacy, while in contrast, the study by Chen et al., indicated that PTX-IONP and PTX had comparable effects on inhibiting U251 cells, but that PTX-IONP had a less potent inhibitory effect on HMC3 cells than pure PTX, indicating that IONP possesses biological safety which could restrict chemotherapy-related toxicity. The results from the study defined PTX-IONP as an effective influencer in tumor suppression via mediating the autophagy-induced ferroptosis [173]. The ferroptosis-based approach using a nanoparticulate system has paved its path toward immunotherapy for cancer as well. Immune checkpoint inhibitors (ICIs), which revive the capability of tumor antigen-specific T lymphocytes to combat cancer, are one of the most exciting methods. Some patients have been able to create an efficient and long-lasting antitumor response because of this immunotherapy. However, only 20% of patients continue to respond to a single ICI. Results by Angulo et al. demonstrated the potential of chemically programmed IONVs as a platform for the development of intrinsically therapeutic vaccines that take advantage of TME reprogramming, as well as the potential clinical utility of tumor susceptibility to iron- and ROS-dependent processes and redox stress during combination immunotherapy [174]. To improve the synergistic effects for cancer therapy, a multifunctional theranostic nanosystem was created by Zhu and the team, using sulfasalazine-loaded nanoparticles and ferric ions. Firstly, SAS and ferric ions were successfully co-loaded onto PEGylated polydopamine (PP) through the interaction of π- π stacking and metal ions to produce Fe (III)PP@SAS NPs enabling dual ferroptosis therapy. Secondly, ferric ions were reduced to ferrous ions triggering the Fenton reaction to produce ·OH radicals. The xCT-GPX4 axis was inhibited by the release of SAS, which dismantled the "shield" that prevents neoplastic cells from undergoing ferroptosis. Second, the release of SAS and Fe (III) from Fe (III)PP@SAS NPs was increased by acid stimulus and laser irradiation, which simultaneously enhanced ferrotherapy. Additionally, ferric ions can be used as a magnetic resonance (MR) imaging agent to visualize the malignant cells, identify the healing alliance and assess the therapeutic impact. As compared to Fe(III) alone, Fe(III)PP@SAS NPs were demonstrated for than 12 h in tumor region in 4T1 bearing tumor mice model due to enhance permeation and retention effect. Regarding this, Fe(III)PP@SAS NPs serve a variety of purposes as a theranostic agent. Overall, the “sword and shield” approach was entitled for multiple beneficial approach, including (a) transition of ferrous ion to ferric ion, (b) tumor visualization by MR imaging, (c) spatiotemporally modified release and (d) promotion of Fenton reaction [175].

Upon the delivery of nanoparticles to the tumor site, cells undergo various biological changes that initiate cell death pathways. Additionally, cancer antigens are released to activate the immune system, particularly inducing cytotoxic T lymphocyte (CTL) responses. The specific cancer antigens liberated are presented by antigen-presenting cells (APCs) through major histocompatibility complexes (MHCs). MHCs play a crucial role in the immune system by enhancing modulatory functions in infected cells during vaccination. They prominently display intracellularly derived proteins and peptides on the cell surface for immune activation [176–178]. The tumor microenvironment constitutes a complex network comprising diverse immune cell types, endothelial cells, pericytes, cancer-associated fibroblasts and other tissue-resident cells. Tumor-associated macrophages (TAMs) account for approximately 30%–50% of the infiltrating cells surrounding cancer cells and are immune cells capable of engulfing particles and releasing cytokines. Investigating the induction of ferroptosis in cancer cells by disrupting the tumor microenvironment through macrophage polarization has been a focal point of research [179–181]. In a study, Fe^3+^ was chelated to form nanoparticles to delivery arginine to the tumor microenvironment. Arginine is identified as a crucial factor in providing support for the energy source and serving as a substrate for the synthesis of proteins and nucleic acids during the processes of CD8+T cell’s activation and progression.

Following accumulation in the tumor, nanoparticles demonstrated the ability to (a) induce immunogenic ferroptosis in cancer cells, leading to maturation of dendritic cells (DCs), initiation of CD8+T cells' infiltration and activation, (b) repolarization of tumor-associated macrophages (TAMs) from M2 phenotype to M1 phenotype, thereby mitigating the tumor immune microenvironment (TIME) and liberating immune-supportive cytokines to sustain the CD8+T cells activity, and (c) release arginine in a way to initiate CD8+T cells proliferation via metabolic adaptation, consequently promoting the immune response [182].

In a reciprocal manner, the restored activated T cells can secrete high levels of interferon-gamma (IFN-γ), enhancing ferroptosis and establishing a closed-loop virtuous circle to potentiate antitumor immunity. This strategy holds significant potential for boosting T cells' activity in anti-cancer immunotherapy. Studies have revealed that iron overload promotes macrophage polarization toward the M1 phenotype through the ROS/acetyl-p53 pathway. This polarization is characterized by an increase in pro-inflammatory cytokines and a decrease in M2 phenotype markers. Notably, M1-type TAMs exhibit a tendency to secrete more H_2_O_2_, promoting the peroxidation of phospholipids containing polyunsaturated fatty acids (PUFA-PLs). Additionally, TAMs release interferon-gamma (IFN-γ), which has been reported to enhance lipid peroxidation and ferroptosis by inhibiting SLC7A11 expression.

Moreover, MHCs, when presented by dendritic cells (DCs) along with tumor antigens, stimulate the activation of immature T-cells in the lymph nodes. This activation triggers tumor-specific CTLs and natural killer (NK) cells, enabling them to regulate cellular events and identify the tumor microenvironments (TMEs) in tumorigenesis. CTLs effectively communicate with T-cell signaling molecules, receptors and MHCs to attract and target tumor cells.

#### Nanozyme

**Fig. 12:**
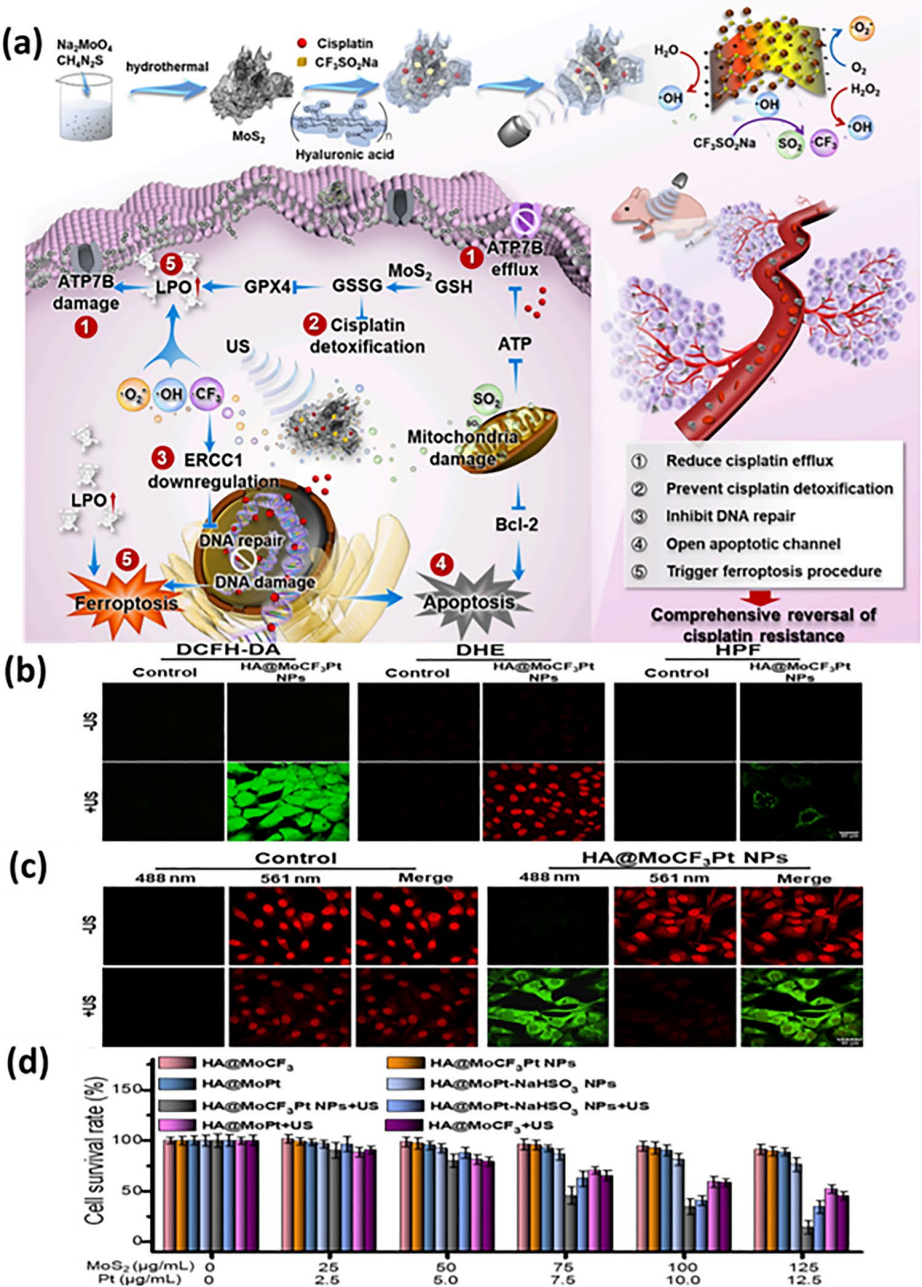
**a** Synthesis of SO2, •CF3 and •OH bearing nanoflower and its inhibiting mechanism for cisplatin resistance cancer under ultrasonic radiation, **b** ROS suppressing effect of nanoflower detected by 2,7-dichlorodihydrofluorescein diacetate (DCFH-DA), dihydroethidium (DHE) and hydroxyphenyl fluorescein (HPH), **c** determination of SO_2_ production capacity coumarin–hemicyanine dye, **d** cell cytotoxicity study of different nanopreparations under US irradiation. Reproduced with permission from Ref. [193]

A group of nanomaterials called nanozyme have enzyme-like properties and can mimic the catalytic processes of natural enzymes [183, 184]. Due to their great stability, tunable activity and affordability, nanozymes are attracting countless attention in the field of biomedicine. The chemical state of a metal ion, pH, glutathione (GSH) level and hydrogen peroxide (H_2_O_2_) are only a few examples of the many variables that can control the enzyme-mimetic actions of nanozymes, showing significant potential for clinical therapy [185]. Nanozymes have been developed for the detection (in vitro), monitoring (in vivo) and therapy of disease by developing a cocktail of specific enzyme-like catalytic activities and physicochemical features [186]. Now, most of the nanozyme are composed of metals and their oxides as they can easily mimic the catalytic electronic redox procedures mediated by natural enzymes [187, 188]. The mimetic activity of enzymes is highly influenced by pH, reducing agents and temperature of the shielded environment. The tumor microenvironment (TME) is widely recognized to have higher redox potential values than normal tissues. These tumor-specific properties can facilitate the ability of nanozymes to operate like enzymes [189–191]. Furthermore, by using various stimuli like light, magnetic field, sonar and heat, nanozymes can be remotely controlled. These variables can be modified as a whole to improve the therapeutic and diagnostic efficacies of various diseases in the field of biomedicine. Due to their great efficiency in generating oxygen radicals (such as •OH and •O_2_) with good stability, piezoelectric materials (such as ZnO, BaTiO_3_, MoS_2_ and BiFeO_3_) have recently been studied in the fields of pollutant degradation, catalysis, tumor eradication and energy transformation. For deep tumor therapy, oxygen radicals could perform more efficiently in a hypoxic tumor environment than ^1^O_2_. It had also been indicated that carbon radicals being more lipophilic than oxygen radicals causes more damage to amino acids present on the organism [192]. To make the ovarian tumor cells responsive toward cisplatin treatment, Ge et al., combined sonodynamic therapy with nanozyme using cisplatin as chemotherapeutic agent, sodium trifluoromethylsulfite (CF_3_SO_2_Na) as a precursor of •CF_3_, hyaluronic acid to develop the outer shell and MoS_2_ nanoflowers (NFs) which can act both as nanoenzyme and sonosensitizer. The nanoflower promotes H_2_O and H_2_O_2_ decomposition generating •O_2_^−^ and •OH, which in turn degrade CF_3_SO_2_Na to form sulfur dioxide (SO_2_) and •CF_3_. Such generation of radicals causes an accumulation of ROS and lipoxygenase to enhance the process of ferroptosis. Using the nanoplatform of cisplatin with US irradiation, the population of SKOV-3/DDP cells declined with no red fluorescence detected after being subjected to confocal microscopy, suggesting degradation of lysosome (potential mechanism behind drug resistance). Furthermore, to confirm the ferroptosis-mediated cell death in vivo*,* western blot technique was performed which discovered downregulation of GPX4 due to extreme rampant redox balance (Fig. 12). On a concluding note, hyaluronic acid-modified nanoframework was successful in the treatment of cisplatin-resistant cancers by employing the another cell killing method called ferroptosis [193]. As nanozymes employ the use of metallic species, they can be employed in tumor imaging as well. In a study by Wang and team, a simple technique was used to successfully design and construct an Avastin (AVS)-modified nanozyme based on superparamagnetic iron oxide (SPIO) for the clinical treatment of triple-negative breast cancer (TNBC). The therapy was, in fact, based on the synergistic approach of starvation therapy and ferroptosis. Starvation therapy works by blocking the vascular endothelial growth factor (VEGF) which suppresses tumor angiogenesis to deduce the supply of nutrients to the malignant cells. Avastin, an anti-VEGF antibody has been established as starvation therapy. The cell line data of MRI and starvation effect on MDA-MB-231 cells after being injected with SPIO and AVS-SPIO were investigated. The results demonstrated a gradual escalation of cellular magnetic signals after 12 h with a significant difference between both groups. The results were further confirmed using the xenograft mice model. On the tenth day of the treatment cycle, the SPIOs and Ava treatment group started to exert tumor-suppressing effects in comparison with the control group. The starvation paired with ferroptosis technique used by the AVS-SPIO group continues to successfully limit tumor development, indicating the superior anti-cancer efficacy of synergistic therapy. Additionally, it was evident from the survival curves of the four distinct treatment groups that AVS-SPIO can significantly increase the overall survival rate. Furthermore, the mice's body temperature and weight were neither negatively impacted by the nanoreactor device. The outcomes demonstrate that GPX4 was substantially inhibited in the AVS-SPIO group, and production of ROS was found highest among all treatment groups. Additionally, the quantitative histological data indicated the synergistic impact of Ava-SPIOs on ferroptosis.

Generally, Fenton reaction is triggered by accumulation of lipoxygenase that actively converts H_2_O_2_ into toxic ·OH. However, low intracellular H_2_O_2_ levels, a limiting supply of iron and unfavorable pH values limit the generation efficiency of hydroxyl radicals, which has a limited therapeutic effect on tumors [194–196].

On this note, photothermal active nanozyme was developed by choosing hollow mesoporous Prussian blue (HMSPB) NPs as a photothermal agent wherein the shell material was composed of phospholipid forming HMSPB-PS. The HMSPB-PS could effectively reach the tumor site and produced photothermal effect under NIR radiation (800 nm) which was employed to destroy cancer cells. Unsaturated lipids in cancerous cells and liposomes can be peroxidized by the iron redox pairs in HMPB. A substantial amount of lipoxygenase produced by photothermal action and lipid peroxidation subsequently leads to robust ferroptosis and finally to tumor ablation. Therefore, photothermal nanoenzyme offers a novel approach by delivering a metal complex including unsaturated lipids and iron redox pairs to imbue lipid peroxidation in a manner distinct from those current techniques, specifically being focused on the Fenton reaction to produce ferroptosis [197]. In another study, Zhang et al. employed caramelization method to develop nanozyme based on gemcitabine (GEM)-loaded MnFe_2_O_4_ carbonaceous (MFC) nanoparticles wherein the loading and entrapment efficiency of GEM was found to be 35.7 mg g^−1^ and 51.6%, respectively. GEM-MFC demonstrated a rapid release of GEM at pH 5 initially for 12 h with sustained release behavior in subsequent hours; however, a slow release was observed at alkaline pH. Because the ester link between Gem and the MFC was broken by the acidic solution, which allowed for a swift release of GEM from GEM-MFC at lower pH levels. Given the outcomes above, GEM-MFC potentially provides GEM in vivo with a pH-responsive release, enabling higher intracellular medication concentrations at the tumor interstitium. The effect of MFC as well as GEM-MFC was visibly investigated by the ROS production effect which was subsequently increased altering the mitochondrial membrane fluidity. An eventual reduced mitochondrial fluidity is highly responsible for its membrane instability causing dramatical change in the membrane of mitochondria. Post-treatment with the nanozyme, the expression of GPX4 was depleted, while those expression of caspase-3 was upregulated demonstrating dual cell death response (apoptosis and ferroptosis). Overall, the salient features of nanozymes unambiguously indicated as biocompatible system with MRI monitoring functionalities along with ferroptosis promotion by exhausting GSH and elevating the level of ROS [198].

#### Magnetic Control of Iron-Based Nanoparticle

Iron-based nanoparticles are also known for their regulated response to an external magnetic field under the influence of permanent magnets. Consequently, their precise positioning can be utilized to accurately determine the site of disease progression. Magnetic nanoparticles share fascinating biomedical applications such as biological catalysis, magnetic separation and targeting, magnetic hyperthermia, drug delivery and photo-responsive treatment, Fe_2_O_3_ NPs [199].

Owing to their remarkable thermal effects in response to an oscillating magnetic field, magnetic Fe_3_O_4_ NPs find extensive application in the realm of magnetic hyperthermia for treating cancer. Additionally, these NPs exhibit catalytic properties akin to peroxidase, functioning as mimic enzymes in cancer therapy through well-established Fenton reactions. In this context, they act as catalysts, converting endogenous hydrogen peroxide (H_2_O_2_) into the highly cytotoxic hydroxyl radical (•OH), leading to the demise of proliferative cells. This innovative approach based on the Fenton reaction, has emerged as a rapidly advancing research area in cancer treatment in recent years, breathing new life into magnetic Fe_3_O_4_ NPs [200–203]. A combination of doxorubicin-loaded polyaspartic acid stabilized iron oxide magnetic nanoparticles and dihydroartemisinin encapsulated polyglutamic acid stabilized iron oxide magnetic nanoparticles not only triggered ferroptosis in triple-negative breast cancer cells but also inhibited PI3K/mTOR pathway [204]. It is interesting to note that apoptosis inducers can also be combined with magnetic iron oxide nanoparticles against cancers. Magnetic iron nanoparticles induce ferroptosis in two ways. Initially, ferrous ion, influenced by hydrolase, degrades into ferric ions. In the subsequent step, these ions induce intracellular iron balance, leading to the release of ROS for the purpose of suppressing tumors [205].

### Mesoporous Silica Nanoparticle

Mesoporous silica nanoparticle (MSN) has gained enormous attention in recent years. Reason is its benefits of tunable and uniform pore size with internal and exterior pores, autonomous surface functionalization and the gating mechanism of the pore opening. These characteristics provide MSNs superior drug encapsulation efficiency, while also being simple to produce employing aqueous sol–gel “chimiedouce” processes. Because of their higher surface charges than anionic or neutral silica particles, cationic silica particles seem to be more cytotoxic and to bioaccumulate more rapidly. In order to demonstrate how charges at the surface of the nanoparticles are a crucial issue to ensure cellular uptake, Davila-Ibáez and colleagues used magnetic silica nanoparticles with DNA connected to the silica network. Particles having a neutral charge, on the other hand, do not seem to internalize in the Caco-2 cells.

More redox systems must be activated by tumor cells to battle ROS stress in order to survive and proliferate. The main redox systems are glutathione (GSH) and thioredoxin (TXN), which use thiol and selenol groups to react with ROS to diminish ROS stress and eliminate them by GSH and ameliorate lipid peroxidation, respectively. Both the GSH and TXN redox systems depend on cysteine (Cys), which serves as the major substrate. This allows the consumption of Cys, which is mostly transported via the system Xc^−^ and predominantly retained in the disulfide form (cystine). Additionally, it has been discovered that breast tumor cells overexpress the xCT protein in comparison with normal cells, providing a high selectivity for tumor therapy. To establish a ferroptosis-based therapy against Xc^−^ system, Li et al. coloaded anti-xCT siRNA with FePt nanoparticle. To create FePt/SiO_2_ (SFP) NPs, FePt NPs were initially produced in situ in the wide pore channels of dendritic mesoporous silicon (SiO_2_) NPs. The siRNA against xCT was then electrostatically loaded into SFP NPs to create siRNA@SFP (sSFP). Interestingly, the SiO_2_ shell successfully prevented the destruction of RNA and unwanted release of FePt during blood circulation. At pH 6.5, 6.3% of the Fe^2+^ ions in SFP NPs were released within 24 h, and more Fe^2+^ ions (equals to 25.7%) were released at pH 5.0. On its contrary, at pH 7.4, only 4.6% of the Fe^2+^ ions were liberated within 24 h. Following an investigation into SFP NPs' pro-oxidative potential, the Fenton property of Fe^2+^ ions was taken into consideration. SFP that had been pre-incubated at pH 7.4 displayed fluorescence signals at 525 nm after being stimulated at 504 nm. The SFP group pre-incubated at pH 5.0 demonstrated the sharpest fluorescence signals, while its pre-incubation at 6.5 demonstrated a comparative stronger pH, which was in unswerving with the liberation of Fe^2+^ ions at various pHs. According to the protein inactivation driven by ROS, the expression of xCT dropped in the SFP group. SiO_2_ alone, however, did not affect the expression of xCT. This nanoplatform thus offered a high level of tumor selectivity with improved therapeutic efficacy confirmed by the subcutaneous xenograft model of breast tumors [206].

A self-eating process called autophagy of the cells enables them to adapt to various stresses such as energy depletion and nutritional starvation while promoting cell survival. In autophagy, encapsulate cellular components which degrade under the action of lysosomal hydrolases to boost the nutrient profile in the cells. Dysfunctional and excessive autophagy, however, might occasionally cause cellular damage. As autophagy has been shown to increase ferroptosis in cancer cells by degrading ferritin, the sensible combination of autophagy with ferroptosis could serve as an effective technique for enhancing the clinical response. Considering this, Yang et al. designed a nanoparticulate system having a dual property of autophagy by trehalose and GSH depleting property of ferroptosis. Manganese oxide-embedded mesoporous silica composite nanoparticles (mSiO_2_@MnO_x_) were thus developed to deliver trehalose (an autophagy inducer) for highly effective tumor therapy via the autophagy-boosted ferroptosis pathway. Employing a simple one-pot sol–gel method, MnO_x_ components were produced in-situ on the surface of mSiO_2_ to trigger ferroptosis via the GPX4-mediated route. To make the pore size and surface area in the acceptable range, the methoxy PEG silane (mPEG) was functionalized on the synthesized nanoparticles developing mSiO_2_@MnO_x_-mPEG nanoparticles (MMM) with high biocompatibility and sufficient water dispersibility that were employed to load trehalose into the mesopores (TreMMM). The cell cytotoxicity of MMM and TreMMM was evaluated on PANC1 and 4T1 cells showing lower cell viability after treatment with TreMMM, which is due to trehalose release in the acidic microenvironment of the cancer cells indicating its anti-cancer potential. It is important to note that cells treated with TreMMM nanoparticles exhibited significantly higher levels of green fluorescence than those treated with MMM nanoparticles. These findings suggest that both TreMMM and MMM nanoparticles may significantly increase LPO levels inside 4T1 and PANC1 cells through GSH depletion, with TreMMM nanoparticles showing a stronger response due to trehalose-induced autophagy. The tumor growth has been successfully retarded by the injection of MMM or TreMMM nanoparticles, but the group treated with free trehalose exhibits no tumor-inhibition activity. The MMM group considerably showed a clear reduction of tumor growth as a result of the efficient accumulation of MMM nanoparticles and consumption of GSH in tumor regions due to MMM-induced ferroptosis. The TreMMM group, which highlights the synergistic effect of trehalose-induced autophagy and GSH scavenge-induced ferroptosis for enhancing ferroptosis, demonstrates the most effective tumor suppression [207]. Thus, such a rational synergistic approach offers an alternative, however, effective cancer treatment strategy.

### Assisted Therapies in Amalgamation with Nanoferroptosis

Nanoferroptosis has also been assisted with various treatment and diagnostic approaches which has been discussed below.

#### Nanoparticle Mediated Sonodynamic-Ferrotherapy Against Cancer

SDT, or sonodynamic therapy, harnesses the synergistic impact of ultrasound (US) and chemical compounds in its treatment approaches particularly through cell death, cavitation, making SDT a minimally invasive option against solid tumors [208–210]. SDT relies on the molecular oxygen, concurrent interaction of low-intensity ultrasound, and US sensitizers to generate ROS. This methodology shares similarities with PDT, where light is used instead of ultrasound to activate sensitizers. An advantage of SDT lies in the deeper tissue penetration achievable with ultrasound compared to the limited reach of light in PDT. This distinction allows SDT to serve as a viable alternative to PDT, as it can effectively reach and produce cytotoxic effects on sensitized tissues situated at greater depths. This indicates the potential for SDT to be more broadly applicable in treating tumor cases and to be clinically employed as a non-invasive solution against malignancies that were previously challenging to access [211–213]. In order to subside the cancerous cells, SDT uses molecular oxygen and sonosensitizers under the influence of low-energy ultrasonic waves. Yu et al., in their research, explored inorganic sonosensitizers owing to diminished phototoxicity and enhanced chemical stability as its counterpart shows long-term accumulation mediated by slower metabolism. Here, ultrasmall Fe-doped zinc oxide nanoparticles were developed as they exhibit specific anticancer effects by undergoing pH-dependent dissolution, particularly in a low-pH environment, releasing Zn^2+^ ions that induce cytotoxicity against cancer cells. Due to their significant exciton binding energy and high redox potential, these nanopreparation generates electron–hole pairs through the transition of electrons from the valence band to the conduction band upon ultrasound irradiation. They can absorb light emitted during ultrasound-induced inertial cavitation enabling zinc oxide nanoparticles to produce ROS in the presence of water or oxygen, achieving anti-cancer sonodynamic therapy (SDT). However, zinc oxide nanoparticles with larger particle sizes exhibit a low degradation rate and prolonged tissue retention following systemic distribution, contributing to inevitable toxicity concerns. Thus, iron-doped zinc oxide nanoparticles as compared to the undoped counterpart had enhanced anti-cancer effect. Such enhanced effect resulted from the induction of ferroptosis, which, under ultrasonic radiation, impeded cell proliferation. Notably, the therapy did not exhibit cumulative toxicity in mice treated with iron-doped zinc oxide nanoparticles. Consequently, the combination of sono-chemodynamic therapy is establishing a new standard in cancer treatment [214].

**Fig. 13:**
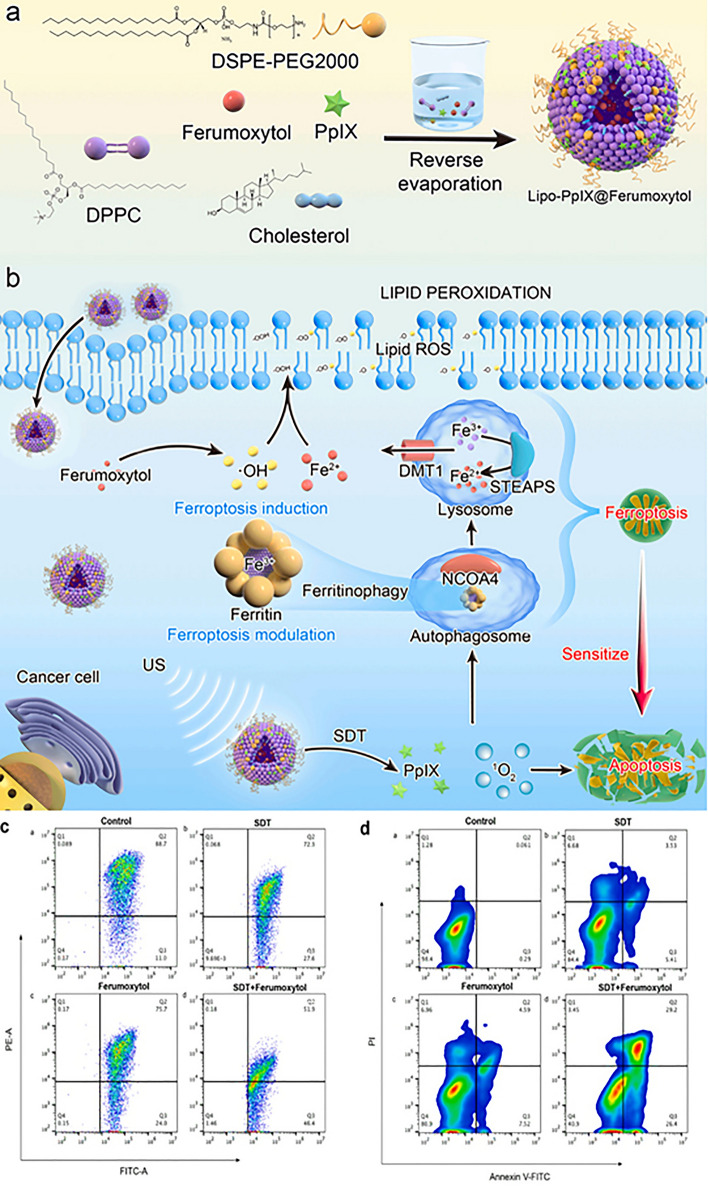
**a** Schematic representation of development method of ferumoxytol-loaded nanoliposome to induce synergistic anti-cancer response (by applying sonodynamic approach) using reverse evaporation method. Ferumoxytol successfully imported iron ions into the cell to stimulate ferroptosis, while photosensitizing agent induced apoptosis by making cancer cells responsive toward therapy, **c**, **d** apoptosis and destruction of 4T1 cells post-treatment with various formulations. Reproduced with permission from Ref. [215]

Sonodynamic therapy is also an approach that involves the generation of ROS by the combination of, molecular oxygen (O_2_), sonosensitizers, and low-intensity focused ultrasound (LIFU). SDT eradicates cancer cells by prompting apoptotic cell death. Unfortunately, due to cancer cells' innate or developed resistance to apoptosis, SDT's ability to induce tumor apoptosis is only a partial success. Considering such checkpoints, Zhou and co-workers aimed to solve the most recurring problem in cancer therapy-resistance to treatment. Liposomes co-loaded with iron supplement ferumoxytol and sonosensitizer protoporphyrin IX were developed to induce two types of regulated cell death (RCD)-ferroptosis and apoptosis which can root out tumor lesions. Ferumoxytol imports iron into the cell which in turn triggers ferroptosis; meanwhile, nanosonosensitizer-enabled SDT controls critical stages of apoptosis and ferroptosis by encouraging cellular-selective autophagy. The prepared nanoliposomes showed a hydrodynamic size of less than 200 nm which is essential for tumor accumulation by archetypal enhanced permeation and retention effect. Western blot analysis was performed to estimate the expression of ferroptosis proteins-GPX4 and acyl-CoA synthetase long-chain family member 4 (ACSL4). The expression of GPX4 is inversely proportional to ferroptosis activity, while ACSL4 is an important biomarker of ferroptosis which acts by sculpting the composition of cellular lipids. According to the data, ACSL4 expression increased by 5.4 times, whereas GPX4 expression decreased by 2.8-fold, indicating that dual-loaded liposomes caused ferroptotic damage to the 4T1 cells influenced by US irradiation. The Lipo-PpIX@Ferumoxytol + US significantly upregulated the expression of pro-apoptotic markers for example B-cell lymphoma protein 2-associated X (BAX) and cleaved caspase 3 (c-CAS3) demonstrating the enhanced apoptotic index of tumor cells due to synergistic influence of synergistic actions of SDT and ferumoxytol. The colony of dead cells was also less in those treated with US + The Lipo-PpIX@Ferumoxytol indicating improved therapeutic effect as compared to US alone. Conclusively, the dual RCD inducing approaches enabled by nanomedicine symbolize a progressing approach in the treatment of therapy-resistant tumor, which raises high hopes for the analyzing additional ferroptosis targeting agents to eradicate refractory cancers (Fig. 13) [215].

#### Amalgamation of Nanoparticle-Based Gene Therapy and Ferroptosis

With a vision to slow down the growth of cancer-causing cells, researchers have shifted toward gene therapy. SiRNA or mRNA-based therapy is novel approaches that either silence, replace or degrade the super active cancer-causing genes. Small interfering RNAs (siRNAs), antisense oligonucleotides (ASOs) and other methods of RNA interference (RNAi) have demonstrated significant efficacy in silencing particular genes. Any target gene of interest can be precisely inhibited by using siRNAs to bind to the mRNA of the intended gene, fragment and destruct it in a sequence-preferential way. SiRNAs-mediated treatment has produced remarkably effective therapeutic responses. They have shown great promise for suppressing genes upregulated in the majority of malignancies [124, 216]. Combination of doxorubicin and siRNA embedded in a nanoparticle reduced the breast tumor size in xenograft mice model suggesting it as a valuable candidate in breast cancer therapy [217]. It is believed that siRNA could mute the gene that regulates the cellular transport of cystine effectively inhibiting intracellular GSH production which is a prerequisite for GSH biosynthesis. The selectivity and potency of siRNA for GSH reduction would be substantially higher than the aforementioned drugs. While considerable advancements have been made with these RNA-based therapies, there are still several critical barriers to the therapeutic application of RNA including polymers and organic linkers and polymers that are unnecessary for organisms to digest and could pose a biosafety risk, the manufacturing processes and elements for nanocarriers are time-consuming and difficult with poor capacity to target tumors. SLC7A11 is a cystine transporter of system Xc^−^ which blocks the cellular uptake of cysteine, thereby affecting the biogenesis of GSH. Hence, a recent study by Huang and team demonstrated an all-in-one nanoparticulate system consisting of iron-siRNA camouflaged with cancer cell membrane. The biomimetic system-induced ROS generation due to Fenton chemistry mediated by iron, while siRNA hampered the GSH synthesis inactivating GPX4 to elevate lipid peroxided accretion. Magnetic resonance imaging (MRI), which enables non-invasive monitoring of the therapeutic efficacy of therapy, was also made possible by the accumulation of Fe in cells [96].

Prostate cancer is one of the deadliest cancers among men with cases of 6%–7% represented castration-resistant prostate cancer (CRPC). A sharp decline in the 5-survival rate from 98% to 30% in such cases has also been observed. First-line medications for metastatic CRPC have so far demonstrated significant results. Examples include the second-generation androgen receptor (AR) inhibitors enzalutamide (Enz) and abiraterone acetate. Unfortunately, the use of these medications has been constrained by drug resistance and poor response. Chen et al. worked on such pitfalls by developing magnetic lipid nanoparticles having ability to induce ferroptosis using 2,4-dienoyl-CoA reductase-1 (DECR1) siRNA (siDECR1) and dihomo-γ-linolenic acid (DGLA). Knockdown or inhibition of DECR1 siRNA might slow the growth of cancer by increasing polyunsaturated fatty acids (PUFAs) and decreasing monounsaturated fatty acids (MUFAs) in prostate cancer cells, thereby inducing ferroptosis in cells by inhibiting GPX4. siDECR1 and DGLA showed synergistic impact displaying potent anti-invasion and antimigration properties among all treated groups [218].

The trend is now shifting toward targeted therapy wherein the therapeutic nanocarrier system is designed in a way to recognize the over-expressed receptors on the tumor cells. Such over-expressed receptors are highly responsible for tumor proliferation and metastasis. Suppression or targeting such receptors is involved in over-coming cancer cell resistance with achievable therapeutic outcomes [25, 124]. Programmed cell death receptor ligand 1 (PD-L1)/PD-1 signaling inhibitors orchestrate promising clinical avenues while having a low level of efficacy. Immunotherapies that target PD-1/PD-L1 checkpoints of the immune system confirmed effective results in the therapy of hepatic cell carcinoma (HCC); however, its monotherapy is limited to a small section of HCC patients. With anticipation to induce the synergistic effect of ferroptosis and suppress PD-1 receptor, Li et al. constructed graphene oxide (GO)-PEI-PEG nanoparticle carrying anti-PD-L1 siRNA in combination with sorafenib (ferroptosis inducer) for targeting HCC. Treatment with siRNA and PD-L1 could elevate the level of ROS potently downregulating GPX4 level causing ferroptosis. Gss and Gsr expression levels are downregulated together with Gpx4 in the tumor areas which shows that GO-PEI-PEG/PD-L1 siRNAs and sorafenib together could immensely produce anti-tumor immunity actions which improve ferroptosis in vivo [219].

#### Nanoparticle-Based Photodynamic Therapy in Association with Ferroptosis

**Fig. 14:**
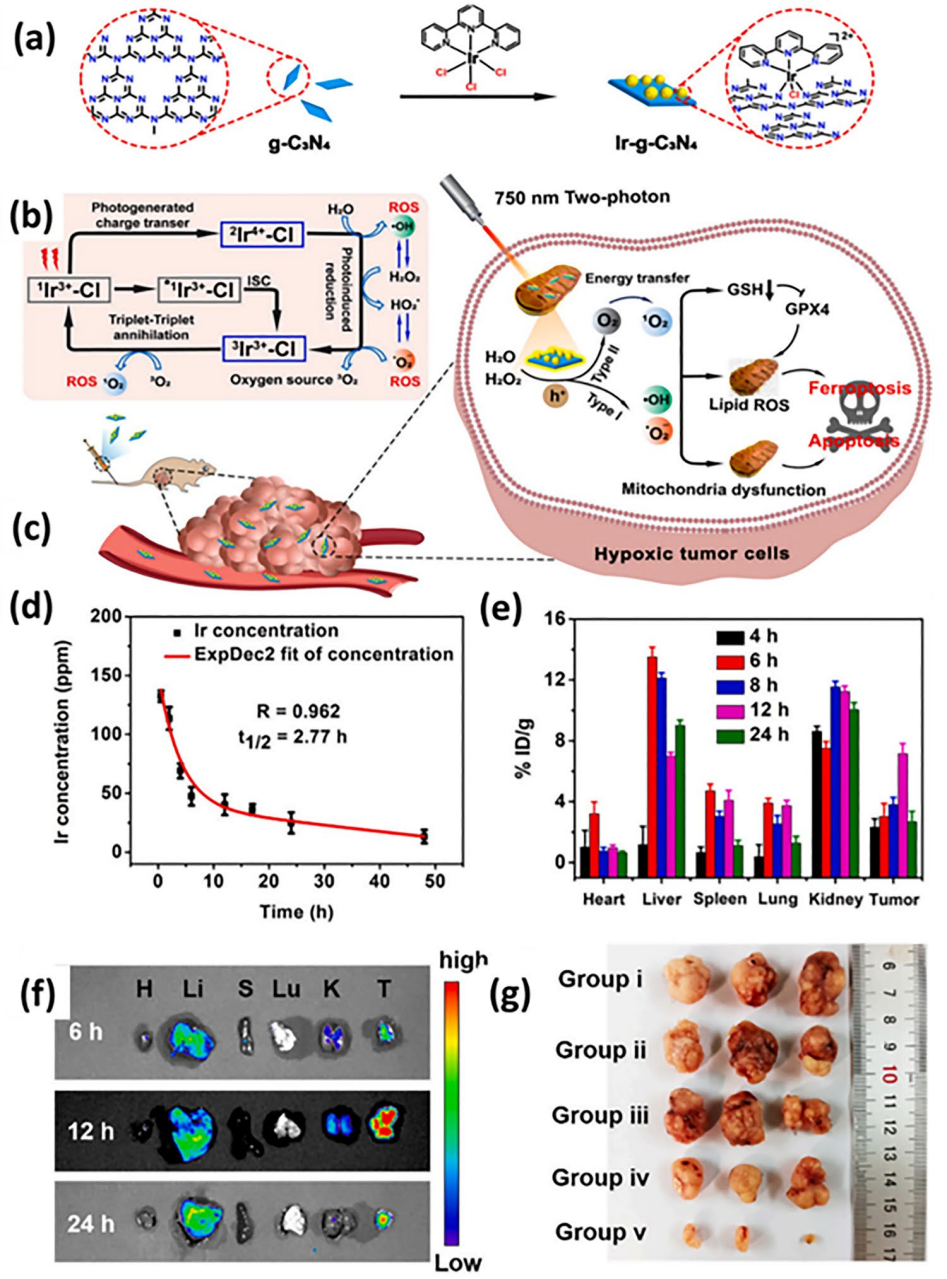
**a** Representation of development of Ir-g-C3N4, **b** regeneration of different kinds of ROS using photosensitizer, **c** representation of mechanism of action of mitochondrial targeting nanosystem responsible for ferroptosis and apoptosis-mediated cell death, **d** blood circulation time post-treatment of Nu/Nu mice with Ir-g-C3N4, **e** biodistribution assay for evaluation of Ir in major organs, **f** luminescence intensity measurement of nanoplatforms at various time interval, **g** digital images of tumor after treatment. Reproduced with permission from Ref. [225]

In the past ten years, PDT has received progressive attention as a means of treating many tumor types, including those of the lung, bladder, stomach, esophagus, head, neck and brain. A photosensitizer (PS) is either systemically or locally administered during a PDT treatment wherein the targeted cells are exposed to the light of a particular wavelength [220, 221]. The PS can catalytically produce lethal ROS, primarily singlet oxygen, upon stimulation. These species are extremely reactive, which causes significant damage to and elimination of the malignant cells and tissue. These compounds, despite their clinical efficacy, have several drawbacks, including inefficient synthesis, low aqueous solubility, poor photostability, poor selectivity, low uptake and sluggish removal from the body, which causes photosensitivity [222–224]. The location and distribution of PS within a neoplastic cell play a crucial role in the efficacy of a PDT agent. According to studies, PSs that are mostly localized within a single-cell organelle exhibit greater therapeutic efficacy than those randomly dispersed throughout the cell. Due to their dual roles as the cell's energy source and the start of numerous types of cell death, the mitochondria had already received a great deal of attention as a subcellular target. Apoptotic cell signals are sent and apoptosis is triggered when a mitochondrion loses its integrity. In a study by, Wei et al., polymeric graphitic carbon nitride nanosheets functionalized with iridium (III) polypyridine complex as a nanophotosensitizer was developed for two-photon-stimulated ferroptosis-assisted photodynamic treatment. These nanosheets served as a polymeric ligand with nitrogen-rich coordination sites. The nanophotosensitizer **(**Ir-g-C_3_N_4_**)** showed increased catalytic capabilities and displayed a synergistic and self-sufficient synthesis of O_2_ from endogenous H_2_O or hydrogen peroxide and the creation of therapeutic substances such as ·OH, ·O_2_ and ^1^O_2_ following irradiation under hypoxic circumstances. In human melanoma cancer (A375) cells, the nanosheets were discovered to be effectively absorbed and localized specifically in mitochondria. The preparation was shown to fragment mitochondria and initiate cell death by combining the apoptosis and ferroptosis pathways when exposed to two photons. The optimal time for PDT was found to be 7.2% at 12 h post-treatment, according to the maximum amount of formulation that was discovered. The luminescence imaging of crucial organs further confirmed the aforementioned findings. The outcomes demonstrated that the tumors treated solely with laser light or with g-C_3_N_4_ nanosheets that were not functionalized in the light both grew asymptotically in a manner similar to the control group. Due to the nanomaterial's capacity to use the overexpressed levels of H_2_O_2_ present inside solid tumors to generate •OH and •O^2−^, which are affecting cellular proliferation, the treatment with Ir-g-C_3_N_4_ in the dark demonstrated a slight tumor growth inhibition effect. On the other hand, group after treatment with Ir-g-C_3_N_4_ and two-photon laser radiation resulted in a marked tumor growth inhibition effect, suggesting high spatial and temporal control as well as the ability to treat deep-seated or large tumors are provided by the two-photon irradiation with NIR light method (Fig. 14) [225].

Redox homeostasis must be tightly controlled in order to increase ferroptosis' effectiveness. Previous studies have attempted to control redox homeostasis, with notable success being obtained with the use of photosensitizer-polydopamine (PDA) as an anti-ROS scavenger, MnO_2_ (consume GSH), and L-buthionine sulfoximine (BSO) (inhibit GSH biosynthesis). As a result, a novel strategy should be planned to simultaneously increase the Fenton reaction level and decrease the GSH level to potentiate ferroptosis [226–228].

Low H_2_O_2_ in tumor cells has also been solved by Luo et al. The regulation of redox homeostasis becomes very important to extend the efficiency of ferroptosis. Cinnamaldehyde (CA) belongs to the class of α, β- unsaturated aldehyde. Due to the conjugation of a double bond in the, α, β-position to the aldehydic functionality of CA, it is capable of undergoing Michael-type addition in form of an electrophilic reaction pathway, which morphs reductive GSH into oxidative GSSG. The balance between GSH and H_2_O_2_ in tumor sites is disrupted as the level of GSH declines, leading to a relatively higher H_2_O_2_ level. Thus, by reducing intracellular reducibility and increasing H_2_O_2_ for the Fenton reaction in the tumor, CA enhances ferroptosis in the malignant cells. Superior anticancer effects have been seen when CA is used as a GSH scavenger to support PDT or PTT. Consequently, CA is an amplifying agent for ferroptosis. Depending upon the chelation reaction between Fe^3+^/Gd^3+^ and dopamine, Luo et al. developed an organic theranostic nanoparticle called FCS/GCS by combining Fe^3+^/Gd^3+^, CA-OH and P-SS-D. The nanoparticles (NPs) were PEGylated by the amphiphilic copolymer (P-SS-D) to augment their circulation time and improve accumulation in tumor tissue. By consuming intracellular antioxidant GSH through the disulfide-thiol exchange reaction, the poly (disulfide) backbone of P-SS-D, which has a high disulfide density and releases drugs into targeted areas, also disturbs the intracellular redox homeostasis in tumor cells. The thioketal (TK) bond in CA-OH releases CA in response to ROS, and the Fe^3+^ catalyzed Fenton reaction increases CA release simultaneously. The abundance of GSH in tumor tissue encourages the conversion of Fe^3+^ to Fe^2+^, which enhances Fenton chemistry and finally ferroptosis. For use in MRI, the released Gd^3+^ can act as a distinct material to show FCS/GCS delivery. After intravenous administration of GCS to 4T1-bearing Balb/c mice, MR imaging showed a gradual increase in the T1 signal intensity within the cancer cells, showing GCS accumulation likely through endocytosis by cancer cells. Particularly, after 4 h of administration, a maximal relative positive augmentation ratio of tumor to the muscle of around 1.46 was attained. Hence it could be suggested that increased ferroptosis was the mechanism by which FCS had therapeutic effects in vivo [229].

**Fig. 15:**
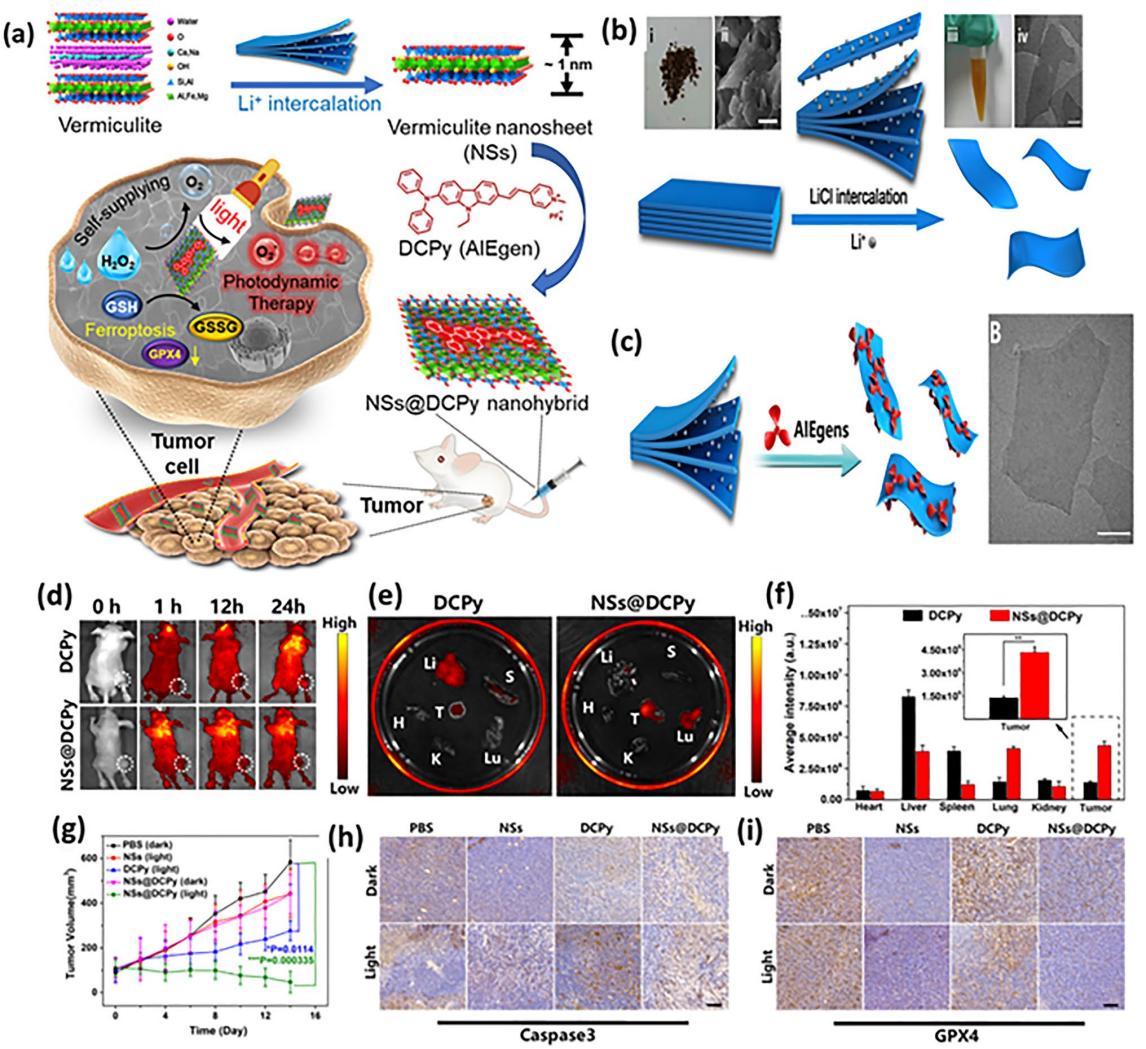
**a** Representation of bonsai-inspired AIE nanohybrid photosensitizer as a key driver in ferroptosis-mediated self-reliable PDT, **b** preparation steps of ultrathin vermiculite nanosystem showing vermiculite in bulk (i), its SEM image (ii) and vermiculite NSs solution (iii), **c** preparation steps of vermiculite NSs@DCPy and its TEM image, **d** In vivo study in mice model showing tumor bio-images with white dotted lines, **e** images of major organs post 24 h of therapy showing spleen (S), heart (H), kidney (K), tumor (T), liver (Li) and lungs (Lu), **f** bio-distribution of NSs@DCPy and DCPy in Balb/c mice model, **g** tumor growth curves, **h** estimation of expression of Caspase 3 and **i** GPX4 by immunohistochemical analysis. Reproduced with permission from Ref. [240]

Because of its lack of invasiveness, rapid healing time and spatiotemporal controllability, PDT has sparked a great deal of interest in the treatment of cancer [230–232]. Photosensitizers, light and oxygen are the three major components of PDT. It relies on light-triggered chemical processes facilitated by photosensitizers that produce deadly ROS from oxygen [231, 233, 234]. It is possible to create effective photosensitizers with superior light stability and a significant Stokes shift using luminogens that display aggregation-induced emission (AIE) features, in particular their capacity to generate singlet oxygen in an aggregated form. The first AIE photosensitizer for PDT was based on TPEIQ, where TPE stands for tetraphenylethylene and IQ stands for isoquinolinium [235–238]. A hypoxic tumor microenvironment (TME), like that found in neoplastic cells, would decrease the effectiveness of photosensitizers because they need oxygen to produce ROS. Given their rapid growth and aberrant blood vessel development, these cells are typically hypoxic, which is made worse by photosensitizer-mediated O_2_ depletion [239]. Tumor hypoxia thus becomes a significant PDT clinical transformation obstacle. On this note, Yu et al. designed AIEgen/vermiculite nanohybrid, a cancer therapy system with a bonsai-inspired O_2_ self-sufficient photoresponsive cancer therapeutic system. Vermiculite from potting soil was employed to create ultrathin nanosheets (NSs), which were then loaded with the AIEgen photosensitizer (DCPy) via electrostatic interaction to construct NSs@DCPy (nanobonsai). A nature-made layered clay known as vermiculite, which is hydrated magnesium–aluminum silicate, is frequently utilized as a nutrient-rich soil for bonsai. Such clays are made up of two identical tetrahedral Al_2_O_3_ and SiO_2_ layers sandwiched by an octahedral Fe_2_O_3_ and MgO layer. The vermiculite-based platform specifically performs the following tasks: While Fe^2+^ catalyzes the Fenton reaction to produce OH, Fe^3+^ catalyzes the TME oxygenation, whereby consumes GSH and liberates O_2_. AIE photosensitizers can be grown on the surface of vermiculite as a nanobonsai, with ultrathin vermiculite nanosheets which deliver O_2_ for the photosensitizers to produce an oxygen-self-sufficient nanosystem which can effectively overcome tumor hypoxia for enhanced PDT. The uptake of NSs@DCPy by hypoxic cells causes the redox pairs (Fe^2+^/Fe^3+^) to produce hydroxyl ion and oxygen through disproportionation reactions of hydrogen peroxide through the Fenton reaction. Incessant production of O_2_ by NSs@DCPy could reduce the hypoxic condition, significantly improving PDT effectiveness. Surprisingly, through iron overload and GSH depletion, the NSs@DCPy was able to cause ferroptosis in tumor cells. The work thus created a ferroptosis-assisted oxygen self-sufficient photodynamic cancer therapy using NSs@DCPy nanohybrid photosensitizers inspired by bonsai (Fig. 15) [240].

### Miscellaneous Nanoparticles with Combinatorial Therapy

Apart from several iron-based nanocarriers, MOF and other discussed nanosized systems, several other nanoparticles have also been investigated which are being discussed here. It has been well observed that nanoparticles possess the ability to overcome multi-drug resistance which has commonly been observed with most chemotherapeutics. Traditional platinum (II) or Pt (II), such as carboplatin or cisplatin, causes systemic toxicity and even acquired resistance that limits their clinical applicability, giving patients with platinum-resistant or refractory relapsed ovarian cancer a dismal prognosis. A group of researchers from China developed 4 different kinds of nanoparticles. Firstly, cisplatin (CIS) and oxaliplatin (OXP) were derivatized by octyl isocyanate to develop 4 prodrugs of Pt (IV) such that the first two prodrugs (Pt (IV)-1 and Pt (IV)-2) were lipophilic having long-chain fatty acids, while the last two had axial maleimide moieties which use thiolmaleimide click chemistry to conjugate with the drug carriers. Being hydrophobic in nature, Pt (IV) 1 and 2 were encapsulated in the human serum albumin nanoparticle forming Abplatin^(iv)^ and Nanoparticle 2 (NP2), while NP3 and NP4 were developed using Pt (IV) 3 and 4 through click chemistry. The cell cytotoxicity assay using the four mentioned nanoparticles demonstrated that NP1 or Abplatin^(iv)^ received top rank in inhibiting the cancer cell growth in which IC50 was found to decrease by 4.47 to 32.91 fold in comparison with CIS. The prodrugs (Pt (IV) 1 and 2) exhibited stronger anti-tumor effect as compared to the parent drugs (CIS and OXP) which is due to elevated cellular uptake generally related to their bionic lipophilic structures. Finally, RNA sequencing demonstrated that, compared to CIS, Abplatin^(iv)^ elevated cell ferroptosis displaying significant antitumor efficacy with limited systemic toxicity [241].

**Fig. 16:**
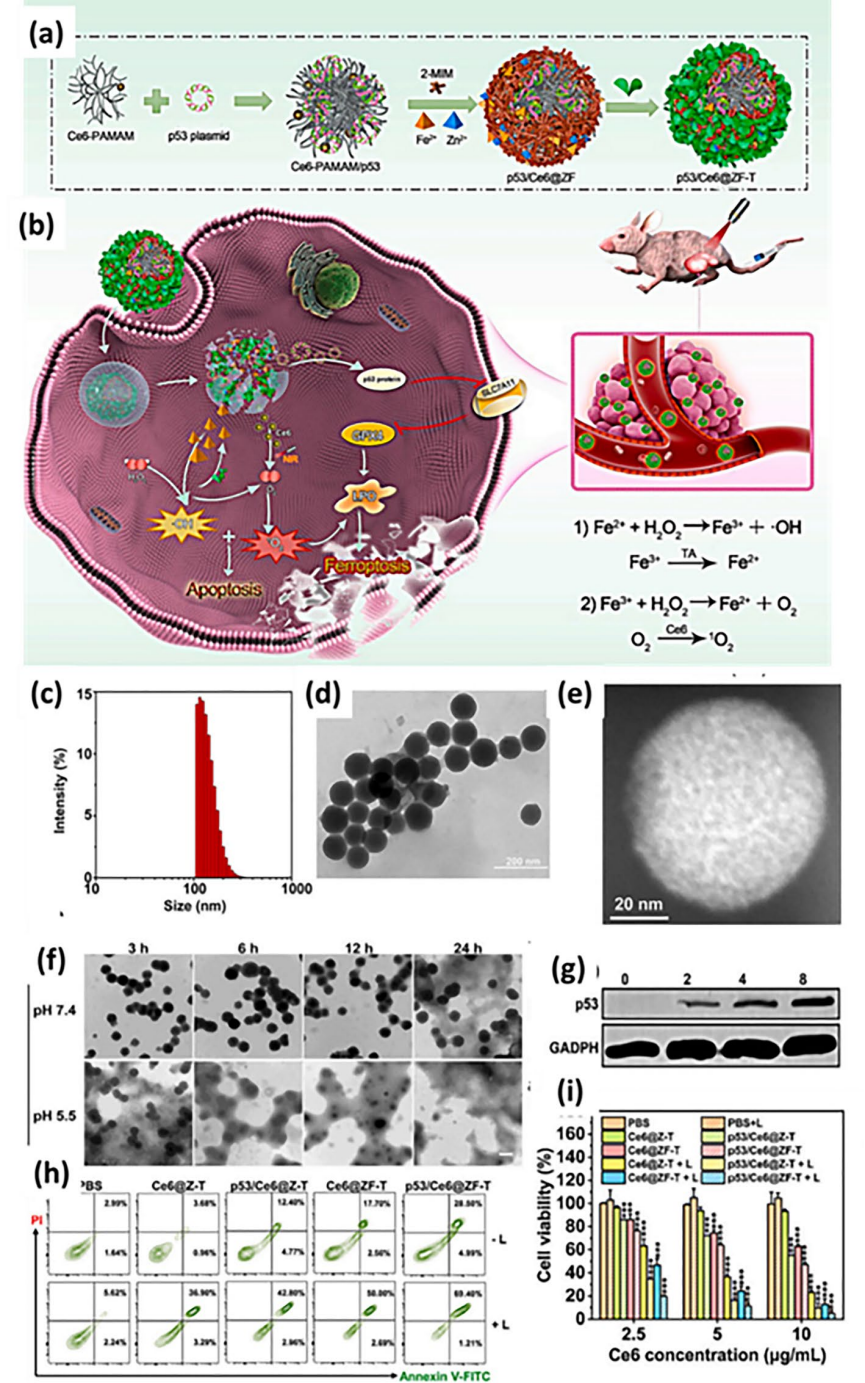
**a**, **b** Preparatory steps of mesoporous zeolite framework comprising of C6-PAMAM and tannic acid, which is then laden with p53 to deplete GSH which is attributed to SLC7A11 inhibition, further causing prominent downregulation of GPX4, **c** size determination using DLS, **d** TEM image, **e** high-angle annular dark-field scanning TEM (HAADF-STEM) image, **f** TEM images of preparation in pH (5.5 or 7.4), **g** estimation of expression of p53 post-incubation of H1299 cells with developed therapy, **h** cell apoptosis study with various treatment groups, **i** cell viability assay. Reproduced with permission from Ref. [246]

In another study a pH-responsive iron-based nanocarrier was constructed using one-pot coordination reaction. The calcium carbonate (CaCO_3_) developed was coated with thin layer of Fe^2+^ and gallic acid with simultaneous encapsulation of succinic acid (SA) and Pt(IV) yielding ^PGF^CaCO_3_ that developed into a hollow structure. The nanosystem was further pegylated (^PGF^CaCO_3_-PEG) to build the capability of pH-responsive drug release, enhance physiological stability, lipid peroxidation and accelerate Fenton reaction. Release quantification results showed 16.1%, 64.0% and 76.6% of Pt(IV)-SA and 8.2%, 22.9% and 46.1%, of Fe^2+^ were released pH 7.4, 6.5 and 5.5, respectively, post-4 h which could be due to fluctuating coordination affinities with CaCO_3_ and GA. In comparison with Pt(IV)-SA and ^GF^CaCO3-PEG, ^PGF^CaCO_3_-PEG exhibited proficient cell killing efficiency, as suggested by the standard cell viability assay. The preparation found capable of enhancing intracellular lipid peroxidation and inhibiting proliferation of tumor cells. Furthermore, the blood circulation results on 4T1 bearing mouse xenograft model have shown to follow two-compartment model showing first and second half-life of 2.65 and 35.3 h, respectively. Importantly, by causing ferroptotic cancer cell death, ^PGF^CaCO_3_-PEG shows significant role in overcoming the therapeutic resistance of standard cisplatin-based therapy [242]. The mode of cellular death via ferroptosis overcomes the treatment resistance those observed by apoptosis pathway, which is due to disturbance in the intracellular redox homeostasis. It is important to keep the factors concerned with ferroptosis. In a nutshell, ROS generation, lipid peroxidation, accumulation of H_2_O_2_ in tumor microenvironment, ^1^O_2_ and ⋅O^2−^ generation, conversion of Fe^3+^ to Fe^2+^ during Fenton reaction are major responsible factors in ferroptosis-mediated therapy. Recent years proclaimed a variety of techniques employed to increase the effectiveness of intratumoral Fenton or related reactions. These techniques include lowering pH levels or raising the level of H_2_O_2_ in the tumor microenvironment, using an external energy field (such as heat, light or ultrasound) to expedite up the reaction rate, or creating high-activity Fenton chemical agents, among others [243–245]. A self-assembled nanoparticle containing Fe^2+^-modified mesoporous zeolitic imidazolate framework-8 conjugated with tannic acid (TA) and cationic chlorin e6-poly (amidoamine) (Ce6-PAMAM) was fabricated by Yu et al., for the treatment of non-small cell lung cancer. The product under NIR laser irradiation showed abundant cytotoxic ROS including singlet oxygen (^1^O_2_) and hydroxyl radical (⋅OH) generated by Ce6-induced PDT and Fe^2+^-interceded Fenton reaction. Additionally, a continuous release of TA guaranteed a constant Fenton chemistry. The preparation in presence of laser irradiation caused stronger depletion of GSH which is attributed to p53-induced SLC7A11 inhibition, further causing prominent downregulation of GPX4. Owing to such effect, an inhibitory effect of laser combined therapy was also observed, while groups treated with PBS in association with laser irradiation showed growth in cancer cells. Immunofluorescence results also confirmed that the substantial cytotoxic effect was due to the synergistic performance of apoptosis–ferroptosis-mediated therapy (Fig. 16) [246].

**Fig. 17:**
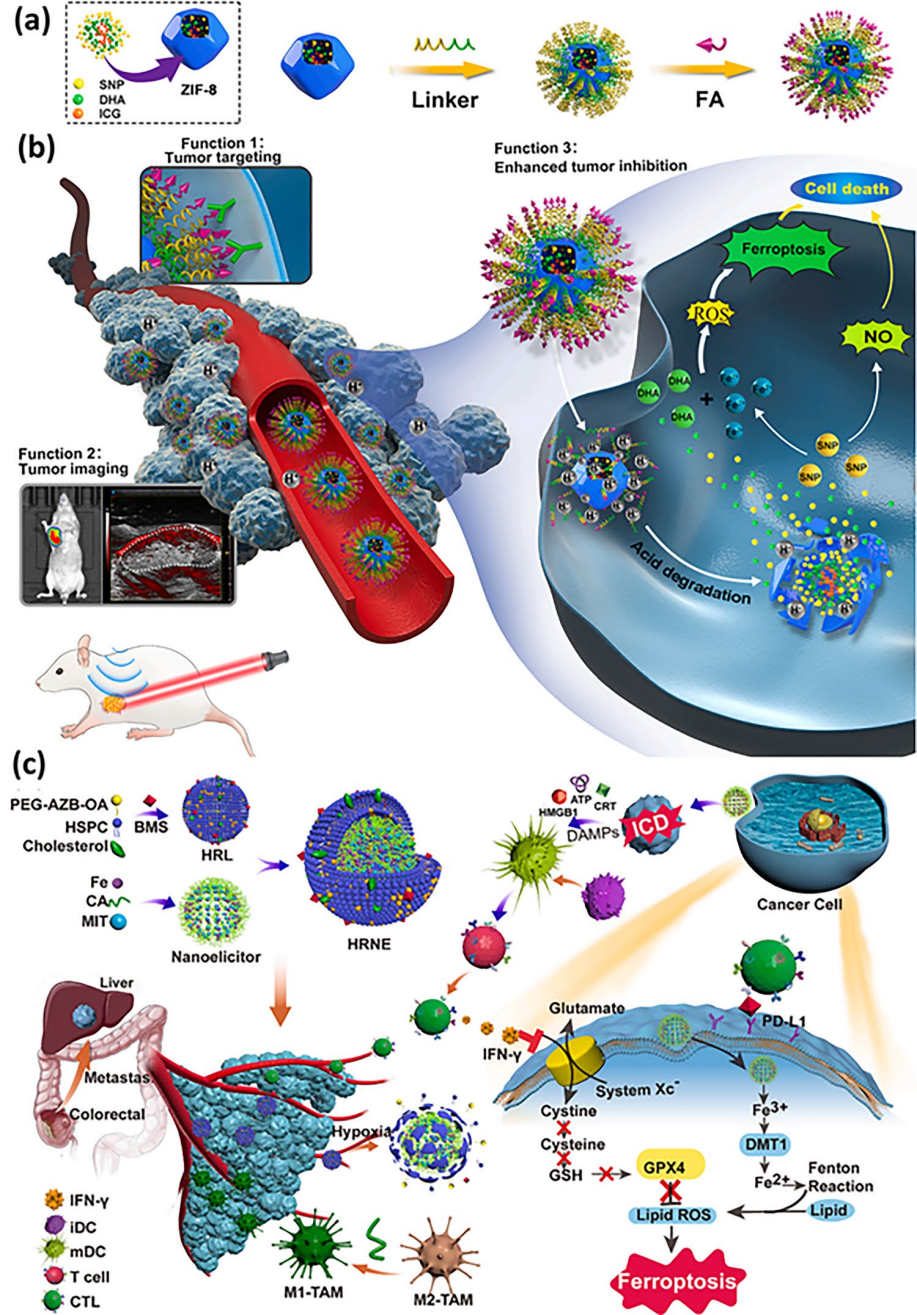
**a** Development process of PEG-functionalized folic acid (FA)-modified zeolite imidazolate frameworks (Zifs) carrying dihydroartemisinin (DHA) as ferroptosis inducer and sodium nitroprusside (SNP) as apoptosis inducer for the treatment of HNSCC; **b** (1) folate receptor enhanced the cellular uptake after binding with FA-functionalized preparation, (2) location of tumor after treatment using imaging tool and (3) elevated anti-cancer effect, **c** representation of development process of MIT-based nanoelicitor to suppress GPX4 expression, enable lipid peroxidation to induce cell ferroptosis [247, 248]

The fact of multi-drug resistance by cancer cells cannot be neglected as most of the cancer-related death is due to non-responsive cell behavior toward therapy. Among them, the treatment of head and neck squamous cell carcinoma (HNSCC) is a major challenge for researchers working in the field of medicine. Apoptosis inhibitor protein expression in cancer cells may be responsible for MDR. Even though targeted medicines like cetuximab (anti-EGFR therapy) have been shown to increase survival, no molecularly targeted treatment has been shown to extend survival in HNSCC. These obstacles show the demand for further cancer therapies. Along these lines, nanotheranostic systems can help suppress the never-ending demand. Zhang et al. aimed to develop a PEG-functionalized folic acid (FA)-modified zeolite imidazolate frameworks (Zifs) carrying dihydroartemisinin (DHA) as ferroptosis inducer and sodium nitroprusside (SNP) as apoptosis inducer for the treatment of HNSCC. Numerous mechanisms of DHA have been examined, among which Fe^2+^ activation is most prominent and highly appreciated owing to its ability in breaking the endoperoxide bridge by the iron ion. However, the major drawback is its short half-life and therefore high dose and repeated injections cause serious side effects. SNP, on other hand, decomposes to form NO and Fe^2+^, thereby potentiating apoptosis and ferroptosis, simultaneously. Both drugs can easily be loaded in Zifs, a kind of MOF, due to its high loading capacity and biodegradability. The targeted preparation showed a high uptake in HN6 cells due to receptor-mediated internalization. With an aim to maximize the ferroptotic effect, DHA/Fe^2+^ was loaded first into the nanoparticle, while SNP was localized at periphery to release prior to DHA. After 24 h of incubation, the release of DHA at pH 4.5 was 88%, while only 6% release was attained at neutral pH. Such a property of nanoparticle is highly required for treatment of cancer to simulate effect in the cellular lysosomal environment. Iron is a demanding element for growth of cancer cells, while free SNP did not induce a positive effect on cell proliferation, suggesting its detrimental response. NO mediates various biological functions including cytoprotective and vasodilation effect which in addition reverses the mechanism of drug resistance. A simultaneous release of both the agents exhibited best anti-cancer efficacy with improved cell deteriorating effect. To deduce the mechanism of cell death, the cells were co-incubated with Ac-DEVD-CHO (APO, an apoptosis inhibitor), α-tocopherol (α-TOC, a lipophilic antioxidant) and the nanoreactor. The cell cytotoxicity was altered significantly suggesting both the methods such as apoptosis and ferroptosis induced the cell death process. Loading of indocyanine green (ICG) helped to tract the system in vivo suggesting the system could overcome resistance, induce clinical efficacy and can be employed as diagnostic agent (Fig. 17a, b) [247]. Apart from treatment resistance, another issue encountered is metastasis of malignant cells. Colorectal cancer (CRC) is a lethal disease with 25% of cases showing liver metastasis. Although radiofrequency ablation has been suggested as a surgical alternative, its efficacy in controlling liver metastases is however inferior to liver resection. CRC, in other terms, is also called as typical immune “cold” tumor which fosters an immunosuppressive tumor microenvironment, meaning they are enriched with immune suppressors [M2 tumor-associated macrophages (M2-TAM)] with infiltrating cytotoxic T lymphocyte. Such a nature of tumor environment is needed to be altered to rebuild an “hot” environment. A self-assembled nanoelicitor comprising mitoxantrone (MIT) and chlorogenic acid (CA) was developed magnetic resonance imaging facility of Fe^3+^ ion. Furthermore, to protect the nanoelicitor from degradation in vivo, a hypoxia-responsive liposomal shell was cloaked on the surface. The aim behind employing MIT is their ability in boosting the activation of T cells and promotes the immunogenic cell death (ICD) of the neoplastic cells in addition to persuading dendritic cell maturation. The herbal extract, CA, endorses M1 polarization of TAM. The in vitro suggested the cytotoxic effect of MIT liberated from the nanoelicitor. To further examine in vitro ICD initiation process of MIT-based nanoelicitor, CT26 cells were coincubated with the preparations, and flow cytometry and CLSM study, based on CRT (calreticulin) which is an ICD biomarker, was performed. The outcomes of the study demonstrated that MIT-based nanoelicitor could elicit the expression of CRT expression. Meanwhile, the expression of GPX4 and lipoxygenase enzyme (FACL4) was reduced post-treatment with developed preparation, validating that ferroptosis generation is the responsible feature involved which amplified the clinical efficacy. A 6.5-fold higher frequency of CD8+T cell responses was elicited in the nanoelicitor-treated mice compared to the saline-treated mice, showing that activated CD8+T and CD4+cells could penetrate tumors and kill tumor cells. It was also discovered that in such groups, significantly higher levels of Granzyme B and IFN-γ expression than in the control group, demonstrating that the immune system had been highly effectively activated (Fig. 17c) [248].

Rapidly increased ionic concentration causes lipid peroxidation, which ultimately results in cell death. However, the continued use of ferroptosis-based cancer treatment in vivo has been constrained by the concurrent systemic toxicity, limited intratumoral accumulation and less expedited reaction. Therefore, targeted ferroptosis techniques have to be optimized to show promise for controlling cancer metastasis [142, 249]. Based on above-described study, other research group, inspired by application of macrophages on cancer treatment developed M1 macrophage extruded nanovesicles (enriched with C–C chemokine-receptor-2 or CCR2) and then loaded with iron-oxide nanoparticles. The equipped nanovesicles possessed exosome mimetic bilayer having size of 100 nm with narrow distribution range. The re-polarization of such M2 macrophages led to H_2_O_2_ build-up, activated T cells via overexpression of T-cell activation factors and improved ferroptosis by synergistically releasing IFN-γ, which again hindered cystine uptake in tumor cells and promoted lipid peroxidation. The synergistic cytotoxic effect and targeted approach employing cellular co-culture techniques and xenograft mouse model were demonstrated both in vitro and in vivo. In order to combat metastasis, the cytomimetic nanoplatform for immune responsiveness and Fe-induced ferroptosis has shown promising results shedding light upon exploration of more synergistic approaches in management of cancer progression [250].

## Conclusion

The treatment of cancer is still far from reality. Indeed, the percentage of patient’s response rate toward therapy is crawling a bit; however, multi-drug resistance and late-stage cancer patients fail to respond toward such conventional therapy. There is a dire need to fasten up the crawling system for cancer therapy. Due to anti-apoptosis and apoptosis evasion brought on by the over-expression of apoptosis protein inhibitors, apoptosis-based techniques have, however, been found to be unable to produce an acceptable clinical effect on cancers in recent years. Cancer cells undergo RAS mutation due to inhibition endogenous apoptosis causing intrinsic cancer resistance. Hence, ferroptosis, another RCD, employs iron-dependent approach of cell killing to conquer apoptotic resistance. The review hence discusses the importance of iron in cancer cell progression with special influence of ferroptosis in conversion of those iron ions to induce Fenton reaction. The molecular basis of ferroptosis through lipid peroxidation, GSH depletion and its substantial inhibitory effect on GPX4 to induce ferroptosis has been discussed. It was also elaborated that by inhibiting system Xc^−^, transport of extracellular cysteine to the cytoplasm will be affected which prevents the formation of GSH and decreases the capacity of cells to fight oxidative stress. This review also shed the light upon chemical basis of nanocarriers in cancer treatment. Nanocarriers could facilitate an easy uptake of ferroptosis inducers coupled with genes or PDT or US irradiation to affect the growth of lethal cells. Recent studies employed MOFs, mesoporous silica nanoparticles, lipid nanoparticles and nanozymes along with miscellaneous nanosystems in ferroptosis-mediated cancer therapy. Such efficient nanosystems can be developed either by self-assembly method or anion-assisted approach. One of the causes of medication resistance is population heterogeneity. Targeting such receptors will have a multifaceted effect since cancer cells express distinct receptors, and the treatment response will vary depending on the patient. With increase in glycolysis, the tumor microenvironment becomes acidic. However, such environment shows variation due to variation in the extent of glycolysis. This in turn affects the iron ion dissolution rate causing variation in induction of ferroptosis. Noteworthy, ferroptosis serves as the main mechanism responsible for regulating the homeostasis of adaptive immune cells. Nevertheless, the excessively high reducing capacity found in immune cells could potentially contribute to the advancement of leukemia T-cells or transformation into B cells. Further research is essential to elucidate the methods for preserving the precise and intricate equilibrium of redox levels within tumor and immune cells in the tumor microenvironment. Additionally, understanding the most effective approach to leverage ferroptosis for inhibiting cancer progression is crucial for future investigations.

In our view, the majority of such therapies are reported in breast cancer treatment, while a few were elaborated on colorectal cancers, head and neck squamous cell carcinoma, ovarian cancer, glioblastoma, and a small number on drug resistance and metastasis. Nanoferrotherapy should be explored in other carcinomas and MDR cases as well. Secondly, biocompatible and cell-grafted nanosystems could be generated which can mimic the biological system to promote its clinical outcomes. To increase the effectiveness of ferroptotic cell death by targeting two or more pathways or regulators simultaneously, additional molecular mechanisms for ferroptosis regulation should be verily identified. Subsequent mechanisms of ferroptosis should be identified with special emphasis on additional ferroptosis inducers to be delivered by biomimetic, biocompatible and biodegradable nanocarriers. The effectiveness of therapy also relies upon the accumulation of ferroptosis inducers inside the cell. Since cancer cells keep reprogramming the structure, treatment fails to reach the desired site or extend itself into the cell. On the other hand, nanotechnology facilitates their successful uptake and fastens the desired therapeutic action. Interestingly, nanomaterial favors to configure the pharmacokinetic profile of the therapeutic agents or ferroptosis inducers. Tumor responsiveness and homologous targeting could easily be achieved. However, the major challenge is synthesis and storage of large-scale nanotherapies. Since quality by design approach is highly applicable to assess the response of material attributes on quality, scientist could employ such approach in development of large-scale therapies. One can quantify the process and material attributes for assessing the desired effect. This in turn will help to monitor the process of ferroptosis both in vitro and in vivo*.*

The outcomes should be paved to next level, i.e., in humans to analyze the potentials more effectively.
